# Identification of quantitative trait loci associated with nitrogen use efficiency in winter wheat

**DOI:** 10.1371/journal.pone.0228775

**Published:** 2020-02-24

**Authors:** Kyle Brasier, Brian Ward, Jared Smith, John Seago, Joseph Oakes, Maria Balota, Paul Davis, Myron Fountain, Gina Brown-Guedira, Clay Sneller, Wade Thomason, Carl Griffey

**Affiliations:** 1 School of Plant and Environmental Sciences, Virginia Tech, Blacksburg, Virginia, United States of America; 2 Eastern Regional Small Grains Genotyping Laboratory, USDA-ARS, Raleigh, North Carolina, United States of America; 3 Eastern Virginia Agricultural Research and Extension Center, Warsaw, Virginia, United States of America; 4 Extension, Virginia Tech, New Kent, Virginia, United States of America; 5 Agricultural Research, USDA-ARS, Raleigh, North Carolina, United States of America; 6 Department of Crops and Horticulture, Ohio State University, Wooster, Ohio, United States of America; Institute of Genetics and Developmental Biology Chinese Academy of Sciences, CHINA

## Abstract

Maintaining winter wheat (*Triticum aestivum* L.) productivity with more efficient nitrogen (N) management will enable growers to increase profitability and reduce the negative environmental impacts associated with nitrogen loss. Wheat breeders would therefore benefit greatly from the identification and application of genetic markers associated with nitrogen use efficiency (NUE). To investigate the genetics underlying N response, two bi-parental mapping populations were developed and grown in four site-seasons under low and high N rates. The populations were derived from a cross between previously identified high NUE parents (VA05W-151 and VA09W-52) and a shared common low NUE parent, ‘Yorktown.’ The Yorktown × VA05W-151 population was comprised of 136 recombinant inbred lines while the Yorktown × VA09W-52 population was comprised of 138 doubled haploids. Phenotypic data was collected on parental lines and their progeny for 11 N-related traits and genotypes were sequenced using a genotyping-by-sequencing platform to detect more than 3,100 high quality single nucleotide polymorphisms in each population. A total of 130 quantitative trait loci (QTL) were detected on 20 chromosomes, six of which were associated with NUE and N-related traits in multiple testing environments. Two of the six QTL for NUE were associated with known photoperiod (*Ppd-D1* on chromosome 2D) and disease resistance (FHB-4A) genes, two were reported in previous investigations, and one QTL, *QNue*.*151-1D*, was novel. The NUE QTL on 1D, 6A, 7A, and 7D had LOD scores ranging from 2.63 to 8.33 and explained up to 18.1% of the phenotypic variation. The QTL identified in this study have potential for marker-assisted breeding for NUE traits in soft red winter wheat.

## Introduction

Wheat (*Triticum aestivum* L.) is among the most widely grown crops in the world and accounts for roughly 20% of the global dietary calories and protein consumed by humans per annum **[1]**. It is therefore crucial to continually improve wheat productivity and quality to meet grain demands in an era of increasing human population. Since the onset of the Green Revolution in the mid-20th century, wheat yield gains in the eastern United States’ soft red winter wheat growing region have been largely attributed to active selection for performance under intensified nitrogen (N) management conditions **[2]**. However, a majority of the N applied to agricultural crops in this region is not harvested and thus subject to loss from the plant-soil system **[3]**. The unharvested N is associated with emissions of nitrous oxides **[4]**, runoff and leaching of nitrate **[5]**, and the degradation of aquatic and terrestrial ecosystems **[6–7]**. Therefore, the development of wheat cultivars that more efficiently take up and utilize applied N provides a means of promoting grower profitability and ensuring environmentally sustainable increases in wheat production.

Genetic improvement of N use efficiency (defined as the ratio of harvested grain per unit N applied; NUE) **[8]** in wheat has been previously reported under a range of N conditions in Europe **[9]**, Central America **[10]**, and the United States **[11]**. While the extent of genetic improvement under differing N conditions varied by study, the authors are generally in agreement that direct selection under multiple N rates will accelerate genetic gains in NUE. This hypothesis was further supported by the detection of significant genotype by N rate (G × N) interactions for grain yield and N traits in recent studies of genotypic variation in wheat **[9, 12–13]**. However, the authors also note that direct selection under multiple N rates may be cost-prohibitive for wheat breeders. In response to this dilemma, Cormier, et al. **[14]** proposed identifying genomic regions associated with N response, known as quantitative trait loci (QTL), to enable more efficient cultivar selection. Through this approach, breeders can efficiently screen germplasm for genetic markers associated with N response to assist in the development of high NUE cultivars.

Previous studies of cereal crops have searched for novel NUE traits and alleles in adapted breeding materials **[15]**, landraces **[16–17]**, and wheat wild relatives **[18]**. While these authors have successfully identified QTL, genes, and genotypes conferring high NUE, additional sources of genetic variation likely still exist within currently unexplored germplasm. This hypothesis has withstood testing in European **[19–22]** and Chinese **[23–26]** wheat and resulted in the successful identification of several QTL associated with N-traits. However, there have been relatively few investigations of N-trait allelic diversity in North American winter wheat, despite known differences in post-domestication selection amongst geographic regions, classes of wheat, and growth habit **[27–29]**. Guttieri, et al. **[11]** and Hitz, et al. **[30]** began the process of dissecting the genetic variation underlying NUE in United States wheat germplasm by conducting genome-wide association studies for N-traits in the central and eastern Unites States, respectively. It is therefore crucial to follow up these investigations using bi-parental mapping populations to validate findings and to enrich potentially rare NUE alleles for subsequent QTL analysis.

Successful QTL mapping for complex traits including NUE is dependent on the selection of suitable parents, evaluation of appropriate population sizes, multi-environment testing, and the development of high-density genetic maps **[31]**. Utilization of genotyping-by-sequencing (GBS) **[32]** derived single nucleotide polymorphisms (SNP) markers aligned to the recently published wheat reference genome **[33]** has the potential to increase marker density and may thus improve the quantity and accuracy of QTL identified for N-related traits. Recently, GBS derived SNPs have been used to detect marker-trait associations underlying qualitative and quantitative traits such as flag leaf architecture **[34]**, disease resistance **[35]**, and yield traits **[36–37]** in winter wheat–resulting in the identification of novel QTL and improved marker quality.

The present study sought to: i) construct a high-density genetic map using GBS derived markers; and ii) identify and validate QTL associated with N-related traits under normal and reduced N conditions. We aimed to detect stable and impactful marker-trait associations for NUE and N-related traits with direct application for marker-assisted breeding programs.

## Materials and methods

### Plant materials

The present study employed a population of 136 RILs, derived from a cross between ‘Yorktown’ and VA05W-151 (herein known as “YT×151”), and a population of 138 DHs, derived from a cross between parental lines Yorktown and VA09W-52 (herein known as “YT×52”). VA05W-151 (PI 665039), a high NUE parent, was derived from the cross of Pioneer ‘26R24’ (PI 614110) / ‘McCormick’ (PI 632691). The other high NUE parent, VA09W-52, is a breeding line derived from a three-way cross, GF921221E16 / McCormick // VA99W-200. Parentage of GF921221E16 is GA83519 / GA85240 // GA861278. Both mapping populations shared a common low NUE parent, Yorktown (PI 667643), which is a product of the three-way cross ‘38158’ (PI 619052) / VA99W-188 // ‘Tribute’ (PI 632689). Parentage of sibling lines VA99W-188 and VA99W-200 is VA91-54-343 (IN71761A4-31-5-48 // VA71-54-147 (CItr 17449) / ‘McNair 1813’ (CItr 15289) / VA91-54-222 (sib of ‘Roane’, PI612958). VA91-54-343 is a sib of VA 92-51-39, which is one of the parents of sibs McCormick and Tribute. The parents used in this study were developed at Virginia Tech for high yield potential under intensive management systems and were assessed for N response and relative NUE in previous investigations of soft red winter wheat **[38–40]**.

### Experimental design

Both populations were grown under rainfed conditions in four Eastern Virginia testing environments (defined as a “site-season”) described in **Table 1**. Testing sites included the Eastern Virginia Agricultural Research and Extension Center near Warsaw, VA in the 2015–2016, 2016–2017, and 2017–2018 winter wheat growing seasons (WR; 37°99' N, 76°78' W) and a commercial production field near New Kent, VA in 2017–2018 (NK; 37°54' N, 76°89' W). Seeds were treated with Raxil MD (triazole, Bayer Crop Science) and Gaucho XT (imidacloprid, Bayer Crop Science) in all testing environments to control diseases and insects, respectively. Foliar pesticides and herbicides were applied throughout the growing season in all environments to further mitigate pest pressure (**S1 Table**). Experimental units, seven-row yield plots that measured 2.74 m long × 1.52 m wide in WR and 4.88 m long × 1.78 m wide in NK, were sown at a seeding rate of 480 seeds m^-2^ to ensure consistent stand establishment.

**Table 1:** Description of environments (Env.) used to test mapping populations.

Location	Season	Env.	Soil series	Soil type	Cumm. Precip.^a^	Cumm. GDD^a^	NO_3_^-^N^b^	NH_4_^+^ N^b^	Total N^c^	Total C^c^
					mm	°C	mg kg^-1^		mg g^-1^	
Warsaw	15–16	16WR	Kempsville	Loam	645	2,905	5.9	1.1	0.5	4.1
	16–17	17WR	Kempsville	Loam	752	2,603	3.9	1.6	0.6	4.4
	17–18	18WR	Kempsville	Loam	1,002	3,379	7.1	1.6	0.5	5.7
New Kent	17–18	18NK	Altavista	Sandy loam	938	3,199	22.0	6.6	1.0	11.2

^a^ Cumulative precipitation and growing degree days (GDD) from planting to harvest.

^b^ Soil nitrate (NO_3_^-^) and ammonium (NH_4_^+^) were determined by analysis of pre-plant KCl filtrates on a Lachat 8500 Flow Injection Analyzer **[41–42]**.

^c^ Total N and organic C determined by combustion analysis.

The two populations utilized a type-2 modified augmented design **[43–44]** in all environments due to limited seed and land availability. The design was comprised of 12 statistical blocks with each block consisting of 30 plots (three rows and 10 columns per block) to facilitate spatial adjustments in all testing environments. Each block consisted of a centrally positioned primary check line (OH08-161-78), two randomized secondary check lines (OH08-172-42 and ‘Sisson’, [PI 617053]) **[45]**, and 12 randomized experimental lines with each check and experimental line being grown under two N rates in adjacent plots. Parental lines were replicated three times per population under each N rate per environment. Wheat lines in each statistical block were grown under high (134 kg N ha^-1^; HN) and reduced (67 kg N ha^-1^; LN) spring N rates that were foliar applied as liquid urea ammonium nitrate split over Zadoks growth stages 25 and 30 **[46]**. Pre-plant N, Phosphorous, potassium, and sulfur were applied according to soil test recommendations prior to planting (**S1 Table**).

### Phenotypic measurements

In the WR site, anthesis date was recorded when half of the anthers had emerged from the florets. Maturity date was recorded in 17WR (where “17” refers to the growing season) and 18WR where it was defined as the date at which when 75% of the peduncles within a plot turned yellow. In all testing environments, plant height was measured before harvest from two random locations within the center rows of each yield plot.

A 1.0 m above-ground biomass (AGBM) sample was cut from the center row of each plot at harvest maturity (grain moisture concentration ≤ 160 g kg^-1^) in all testing environments. Biomass samples were oven dried at 60°C for 72 hours and weighed to estimate AGBM yield for all experimental and check lines. Samples were then threshed to estimate harvest index (calculated as the ratio of grain biomass per unit of total AGBM) by separating grain from straw and chaff tissue and recording their respective weights. Grain and straw tissues were ground through a 2 mm sieve and homogenized to estimate tissue N concentration via combustion analysis using a Vario Max Cube elemental analyzer (Elementar Analysensysteme, Hanau, Germany). Results of the combustion analysis enabled calculation of grain N concentration, NUE, N uptake efficiency (NUpE), and N utilization efficiency (NUtE). Moll, et al. **[8]** defined the terms NUpE and NUtE as quotients of NUE where NUpE is calculated as the ratio of aboveground N at harvest per unit of N applied and NUtE as the amount of grain produced per total aboveground N in the plant at harvest. Plots were combine harvested (Wintersteiger Classic; Wintersteiger, Ried, Austria) in all testing environments at harvest maturity and adjusted to 0 g moisture kg^-1^ to determine grain yield.

### Statistical analysis

An analysis of variance (ANOVA) was performed for the replicated parents using the lme4 package **[47]** in the R statistical computing environment **[48]**:

$$Y_{ijkl}=\mu +G_{i}+N_{j}+E_{k}+R_{l}\left(E_{k}\right)+GN_{ij}+GE_{ik}+NE_{jk}+GNE_{ijk}+\varepsilon_{ijkl}$$

Where the trait response (*Y*_*ijkl*_) is a function of the overall mean (*μ*), the fixed effect of the *ith* wheat line (*G*_*i*_), the fixed effect of the *jth* N rate (*N*_*j*_), the random effect of the *lth* replication (*R*_*l*_) nested within the *kth* environment (*E*_*k*_), the interactions of the *ith* wheat line with the *jth* N rate and the *kth* environment (*GN*_*ij*_ and *GE*_*ik*_), the interaction of the *jth* N rate and the *kth* environment (*NE*_*jk*_), their 3-way interaction (*GNE*_*ijk*_), and the residual error (*ε*_*ijkl*_). This was followed by an ANOVA for parental lines within environment as the effects of testing environment and its interactions were significant for most traits. Means comparisons were conducted using least significant differences for single effects and their interactions. Following the ANOVA, a bivariate correlation analysis calculated Pearson’s correlation coefficients using trait means for progeny in each population under LN and HN conditions.

### Genotyping

Genomic DNA was isolated from seedlings of RILs, DHs, and parents at the three-leaf stage using an LGC 218 Genomics Oktopure^™^ robotic extraction platform with sbeadex^™^ magnetic microparticle 219 reagent kits at the USDA-ARS Eastern Regional Small Grains Genotyping Center (Raleigh, NC, United States). Three technical replicates of each parent and single replicates for RILs and DHs were submitted for genotyping. Genotyping-by-sequencing was performed using an Illumina HiSeq 2500 following the protocol of DNA digestion with the restriction enzymes *PstI* and *MspI* **[49]**. Sequence reads were aligned to the ‘Chinese Spring’ v1.0 reference genome **[33]** using the Burrows-Wheeler Aligner v0.7.17-r1188 **[50]**. TASSEL-GBS v5 **[51–52]** was used to perform SNP calling, and to generate consensus calls for replicated parent samples. Resulting genotypic data was then filtered to retain SNPs with less than 20% missing data frequencies and less than 5% heterozygous calls using VCFTools v0.1.16 **[53]** and BCFTools v1.9 **[54]**. SNP calling and filtering was performed on each population separately. Missing data was not imputed and only SNPs with differing parental homozygous calls were retained. The remaining genomic DNA from each population was used to amplify polymorphic markers from a set of 116 SNP and simple sequence repeat (SSR) markers used in routine screening of the uniform and regional breeding nurseries (**S2 Table**).

### Construction of genetic maps and detection of QTL

Polymorphic markers were used to construct high-density linkage maps for the YT×151 and YT×52 populations. The SNP calls were converted to an ABH parent-based format for the construction of linkage maps in JoinMap v. 4.0 **[55]**. Within JoinMap, map distance was determined using the Kosambi mapping function **[56]** and linkage groups were constructed based on a minimum logarithm of odds (LOD) threshold value of 3.0 and retained all markers. IciMapping v. 4.1.0 **[57]** was used to identify QTL in both populations using composite interval mapping for traits under LN and HN conditions in each environment. The critical threshold to declare a QTL significant (P < 0.05) was based on 1000 permutations **[58]** with a minimum LOD value of 2.5 for traits within each N-environment. Linkage maps were drawn using MapChart v. 2.2.3 **[59]**.

## Results

### Phenotypic variation and trait correlations

**Fig 1:**
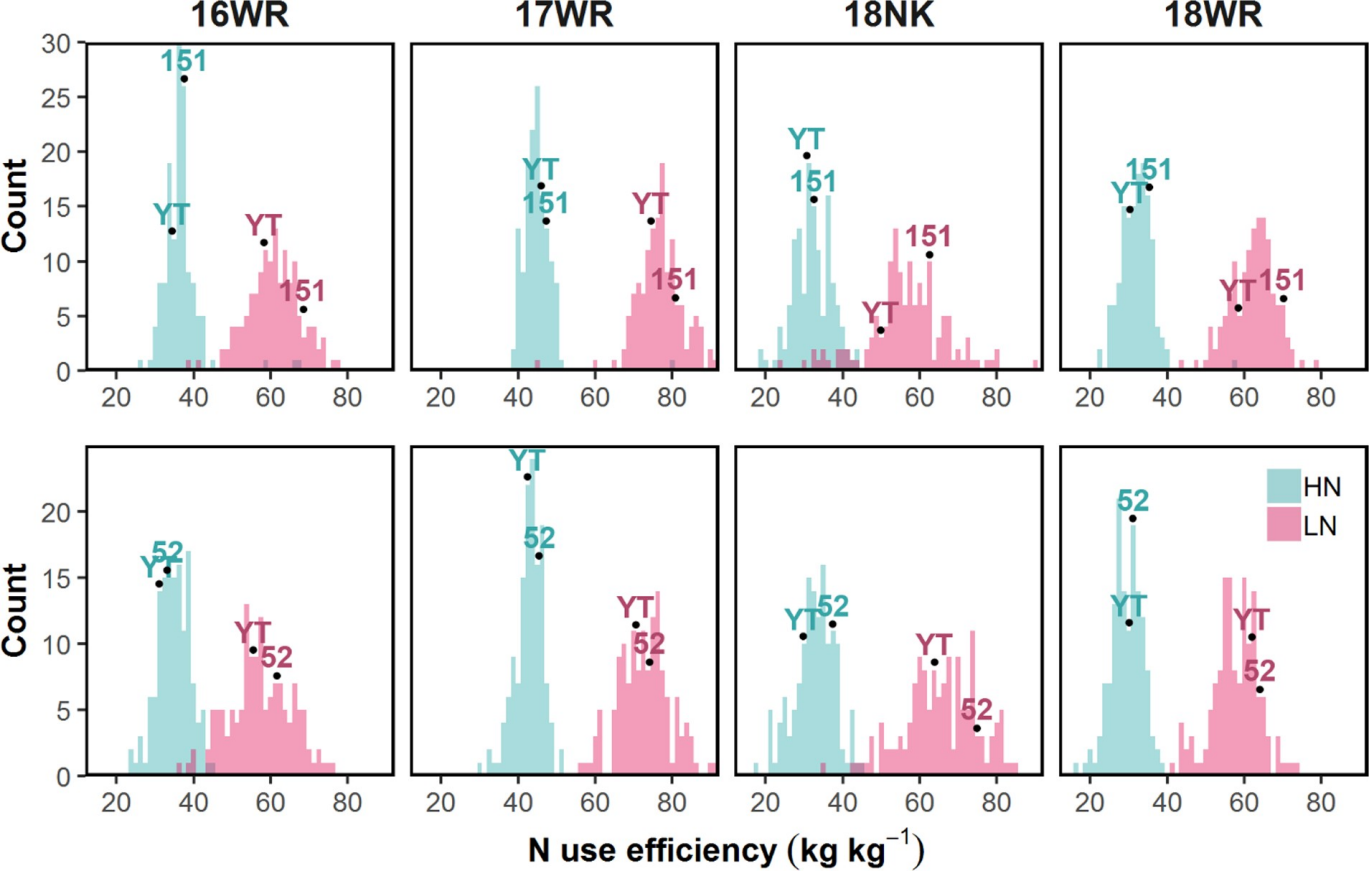
**Nitrogen use efficiency histograms of wheat parents and progeny from the Yorktown × VA05W-151 (top) and Yorktown × VA09W-52 (bottom) populations grown under low (LN) and high (HN) N rates in each testing environment.** Parent means at each N rate are shown for Yorktown (YT), VA05W-151 (151), and VA09W-52 (52). Detailed statistical analysis of parents and progeny in **S5 and S6 Tables**.

The ANOVAs for the replicated parental lines, N rates, and their interactions within each environment are shown in **S3 and S4 Tables**. Variance of parents and summary statistics of progeny for all traits at each N-environment for the YT×151 and YT×52 populations are reported in **S5 and S6 Tables,** respectively. Significant phenotypic variation was observed in the two populations under each N rate for a majority of the studied traits. All progeny reached anthesis over a seven and 10-day period in the YT×151 and YT×52 populations, respectively. The parents in both populations expressed similar plant heights, anthesis dates, and maturity dates within each testing environment despite variation at the Ppd-D1 locus in the YT×52 population (**S2 Table**). Parents of the YT×151 population differed significantly in NUE under LN supplies in all testing environments, while their NUE was not statistically different under the HN rate (**Fig 1 and S5 Table**). The YT×52 population expressed a similar trend but the parents only differed significantly under the LN supply in the 18NK environment (**S6 Table**).

In both populations, NUE was strongly and positively associated with AGBM, harvest index, and N uptake efficiency under LN and HN rates (**Tables 2 and 3**). Within the YT×52 population, NUE was significantly correlated with grain N concentration (LN: r = -0.32, P < 0.001; HN: r = -0.32, P < 0.001) and NUtE (LN: r = -0.31, P < 0.001; HN: r = -0.38, P < 0.001). Under HN rates, lodging was associated with grain yield (r = -0.40, P < 0.001), grain N concentration (r = 0.24, P < 0.01), harvest index (r = -0.37, P < 0.001), NUtE (r = -0.21, P < 0.05), and plant height (r = 0.23, P < 0.01) in the YT×151 population, while lodging was only associated with plant height (r = 0.44, P < 0.001) in the YT×52 population. Nitrogen uptake efficiency was negatively correlated with NUtE under LN and HN rates in both populations. However, this association was greater for RILs in the YT×151 population under LN (r = -0.78, P < 0.001) and HN (r = -0.82, P < 0.001) rates compared to DHs in the YT×52 population under LN (r = -0.52, P < 0.001) and HN (r = -0.47, P < 0.001) conditions.

**Table 2:** Trait correlations under low (bottom left) and high (top right) N rates in the Yorktown × VA05W-151 wheat population. Pearson’s correlation coefficients calculated from means of the 136 RILs over testing environments.

	GY^a^	GNC	AGBM	HI	NUE	NUpE	NUtE	AD	HGT	LDG
GY		-0.23**	0.60***	0.48***	0.99***	0.36***	0.16	0.02	0.02	-0.40***
GNC	-0.12		-0.00	-0.28**	-0.23**	0.31***	-0.51***	0.10	0.07	0.24**
AGBM	0.74***	0.01		-0.39***	0.60***	0.66***	-0.32***	-0.09	0.20*	-0.10
HI	0.46***	-0.25**	-0.15		0.48***	-0.29***	0.53***	0.10	-0.19*	-0.37***
NUE	0.98***	-0.13	0.80***	0.45***		0.35***	0.15	0.02	0.02	-0.40***
NUpE	0.45***	0.30***	0.58***	-0.10	0.46***		-0.82***	0.04	0.26**	-0.01
NUtE	0.06	-0.47***	-0.16	0.38***	0.08	-0.78***		-0.04	-0.26**	-0.21*
AD	-0.03	-0.05	-0.02	-0.01	0.01	0.06	-0.05		0.08	-0.11
HGT	0.18*	0.07	0.29***	-0.20*	0.14	0.28***	-0.17	0.11		0.23**
LDG	0.00	0.15	0.09	-0.09	0.01	0.11	-0.11	-0.05	0.31***	

^a^ Trait abbreviations for grain yield (YLD), grain N concentration (GNC), above-ground biomass (AGBM), harvest index (HI), N use efficiency (NUE), N uptake efficiency (NUpE), N utilization efficiency (NUtE), anthesis date (AD), plant height (HGT), and lodging (LDG).

* Significant at the 0.05 probability level.

** Significant at the 0.01 probability level.

*** Significant at the 0.001 probability level.

**Table 3:** Trait correlations under low (bottom left) and high (top right) N rates in the Yorktown × VA09W-52 wheat population. Pearson’s correlation coefficients calculated from means of the 138 DHs over testing environments.

	GY^a^	GNC	AGBM	HI	NUE	NUpE	NUtE	AD	HGT	LDG
GY		-0.33***	0.49***	0.34***	0.99***	0.46***	0.36***	0.11	0.09	-0.01
GNC	-0.32***		-0.10	-0.16	-0.32***	0.21*	-0.47***	-0.19*	-0.28***	0.04
AGBM	0.70***	-0.12		-0.24**	0.49***	0.71***	-0.01	0.05	0.06	0.01
HI	0.50***	-0.31***	-0.24**		0.34***	-0.16	0.42***	-0.18*	-0.12	-0.07
NUE	0.99***	-0.32***	0.70***	0.50***		0.46***	0.38***	0.09	0.08	-0.02
NUpE	0.48***	0.36***	0.50***	0.03	0.48***		-0.47***	-0.03	-0.10	-0.01
NUtE	0.30***	-0.45***	0.12	0.29***	0.31***	-0.52***		0.08	0.18*	-0.04
AD	0.03	-0.11	0.13	-0.14	0.02	-0.07	0.07		0.42***	0.04
HGT	0.21*	-0.10	0.29***	-0.06	0.21*	0.05	0.10	0.39***		0.44***
LDG	0.13	0.05	0.09	0.04	0.12	0.12	-0.05	0.05	0.30***	

^a^ Trait abbreviations for grain yield (YLD), grain N concentration (GNC), above-ground biomass (AGBM), harvest index (HI), N use efficiency (NUE), N uptake efficiency (NUpE), N utilization efficiency (NUtE), anthesis date (AD), plant height (HGT), and lodging (LDG).

* Significant at the 0.05 probability level.

** Significant at the 0.01 probability level.

*** Significant at the 0.001 probability level.

### Linkage map construction

After filtering for low-quality markers, linkage maps comprised of 3,918 markers spanning 2,962.8 cM in the YT×151 population and 3,147 markers spanning 2,491.7 cM in the YT×52 population (**Table 4**). Within the YT×151 population, chromosome 6B had the lowest coverage (7.8 cM) despite being comprised of 92 genetic markers, while 7A had the greatest coverage (238.6 cM) and consisted of 366 markers. The YT×52 population’s shortest linkage map on chromosome 4D consisted of 12 markers spanning 17.0 cM and its longest was on chromosome 5A having 249 markers that spanned 227.4 cM. Marker densities ranged from 4.18 (3D) to 0.08 (6B) cM per marker for the YT×151 and from 2.69 (3D) to 0.32 (1A) cM per marker for the YT×52 population. The YT×151 linkage map had gaps greater than 30.0 cM on 3B, 3D, and 4A, while the longest gap in the YT×52 population was on 5D (28.0 cM).

**Table 4:** Description of the genetic linkage maps for the Yorktown × VA05W-151 and Yorktown × VA09W-52 wheat populations.

Chromosome	Yorktown × VA05W-151				Yorktown × VA09W-52			
	No. of markers	Coverage	Average Spacing	No. of linkage groups	No. of markers	Coverage	Average Spacing	No. of linkage groups
		cM				cM		
1A	67	68.0	1.01	3	202	65.3	0.32	1
1B	264	132.6	0.50	2	152	107.6	0.71	3
1D	111	126.1	1.14	2	74	91.2	1.23	2
2A	367	218.3	0.59	3	255	117.3	0.46	3
2B	134	38.0	0.28	2	281	145.8	0.52	2
2D	138	160.6	1.16	1	61	97.3	1.60	2
3A	216	175.2	0.81	3	240	184.3	0.77	1
3B	300	218.0	0.73	2	299	161.3	0.54	2
3D	48	200.4	4.18	4	30	80.8	2.69	2
4A	268	188.9	0.70	3	65	128.6	1.98	2
4B	159	110.4	0.69	2	108	92.5	0.86	1
4D	30	100.5	3.35	2	12	17.0	1.42	1
5A	180	128.1	0.71	3	249	227.4	0.91	1
5B	273	166.1	0.61	1	245	154.8	0.63	2
5D	70	146.3	2.09	3	56	150.7	2.69	2
6A	325	175.3	0.54	1	183	93.7	0.51	2
6B	92	7.8	0.08	2	65	59.4	0.91	2
6D	28	60.6	2.16	2	70	78.8	1.13	2
7A	366	238.6	0.65	3	241	173.6	0.72	3
7B	372	204.6	0.55	3	187	129.3	0.69	2
7D	110	98.4	0.89	3	72	135.0	1.88	2
A genome	1,789	1,192.4	0.72	19	1,435	990.2	0.81	13
B genome	1,594	877.5	0.49	14	1,337	850.7	0.69	14
D genome	535	892.9	2.14	17	375	650.8	1.81	13
Total	3,918	2,962.8	1.12	50	3,147	2,491.7	1.10	40

### QTL in the YT×151 population

The YT×151 linkage map was employed to detect QTL with a LOD score greater than 2.5 for 11 traits. A total of 66 QTL were identified for traits in one or more N-environments (**S7 Table**) of which 12 QTL were reproducible in two or more N-environments (**Table 5**). The combined 66 QTL mapped to the A (24), B (17), and D (25) genomes. Reproducible QTL mapped to chromosomes 1D, 2B, 3B, 4A, 5A, 6A, and 7D, and individually explained 4.7 to 27.5% of the phenotypic variation. The LOD values among reproducible QTL ranged from 2.61 to 21.85 for *QAgbm*.*151-2B* in 17WR-LN and 18WR-HN, respectively. The QTL on 3B, *QHi*.*151-3B*, was located within 11 cM of the SNP IWA4755 **[60–61]**, conferring resistance to Fusarium head blight (FHB, caused by the fungal pathogen *Fusarium graminearum*) that was identified in the wheat cultivar ‘Bess,’ PI 642794 **[62]**. The reproducible QTL *QAgbm*.*151-2B*, *QNue*.*151-1D*, *QNue*.*151-7D*, and *QMd*.*151-5A* were not located near any of the 116 haplotype markers with known trait associations.

**Table 5:** Quantitative trait loci (QTL) associated with N and agronomic traits in 2 or more N-environments in the Yorktown × VA05W-151 wheat population.

Trait	QTL	Chr.^a^	N-Env.^b^	Pos.	Left marker	Right marker	LOD^c^	PVE^d^	Add^e^
								%	
AGBM^f^	*QAgbm*.*151-2B*	2B	17WR-HN	2	S698090811	S699106811	2.86	4.7	-21.19
			17WR-LN	1	S699106811	S701094516	2.61	6.2	-22.01
			18WR-HN	3	S687334185	S683005457	21.85	27.5	-134.54
	*QAgbm*.*151-6A*	6A	17WR-LN	80	S421525657	S446092289	4.91	11.7	31.12
			18NK-LN	72	S549302316	S508845927	4.84	13.5	85.26
HI	*QHi*.*151-3B*	3B	16WR-LN	71	S418440403	S452107771	3.56	13.1	0.01
			18WR-LN	80	S58771568	S520444036	2.76	7.3	-0.01
	*QHi*.*151-4A*	4A	18NK-HN	37	S423586538	S7441669	3.15	10.0	-0.01
			18WR-HN	49	S104673741	Fhb_4A_Neuse	4.75	14.6	-0.02
NUE	*QNue*.*151-1D*	1D	17WR-HN	67	S31421492	S52583851	7.40	17.1	1.25
			17WR-LN	67	S31421492	S52583851	8.33	18.1	2.42
	*QNue*.*151-4A*	4A	18NK-HN	24	S17017927	S565759788	4.67	13.7	-1.87
			18WR-HN	35	S137557861	S387080461	4.33	13.2	-1.46
			18WR-LN	24	S17017927	S565759788	3.37	10.3	-1.71
	*QNue*.*151-6A*	6A	17WR-LN	81	S426173022	S141861675	4.85	9.3	1.77
			18NK-LN	74	S508845927	S473574940	3.18	8.7	3.86
	*QNue*.*151-7D*	7D	16WR-HN	66	S171304706	S176567249	3.52	12.6	1.07
			16WR-LN	67	S176567249	S213740699	2.80	7.5	1.78
			17WR-HN	48	S121210261	S153640051	5.25	10.9	0.99
			17WR-LN	48	S121210261	S153640051	2.86	5.3	1.29
NUpE	*QNupe*.*151-4A*	4A	16WR-HN	63	S104673741	Fhb_4A_Neuse	3.91	12.3	-0.18
			16WR-LN	63	S104673741	Fhb_4A_Neuse	10.84	12.7	-0.59
NUtE	*QNute*.*151-4A*	4A	16WR-HN	65	S581951171	S583968823	3.61	11.2	-2.74
			16WR-LN	62	S104673741	Fhb_4A_Neuse	10.95	20.0	-6.51
			18WR-HN	49	S104673741	Fhb_4A_Neuse	3.02	9.5	-1.55
MD	*QMd*.*151-5A*	5A	17WR-HN	7	S511963634	S544461754	4.55	11.9	-0.52
			17WR-LN	9	S511963634	S544461754	5.63	11.2	-0.58
	*QMd*.*151-6A*	6A	17WR-HN	84	S141861675	S117072369	6.91	15.8	0.58
			17WR-LN	84	S141861675	S117072369	7.85	14.8	0.65

^a^ Chromosome (Chr.).

^**b**^ Numbers indicate years 2015–2016 (16), 2016–2017 (17), and 2017–2018 (18); letters indicate locations Warsaw (WR) and New Kent (NK); low (LN) and high (HN) N rates within environment.

^c^ Logarithm of odds.

^d^ Percentage of phenotypic variation explained by the QTL.

^e^ Level of additivity. A positive sign indicates that alleles from Yorktown increased the trait value and a negative sign indicates that alleles from VA05W-151 increased the trait value.

^f^ Trait abbreviations for above-ground biomass (AGBM), harvest index (HI), N use efficiency (NUE), N uptake efficiency (NUpE), N utilization efficiency (NUtE), and maturity date (MD). Grain yield was excluded from the results as its QTL were identical to those found for NUE.

**Fig 2:**
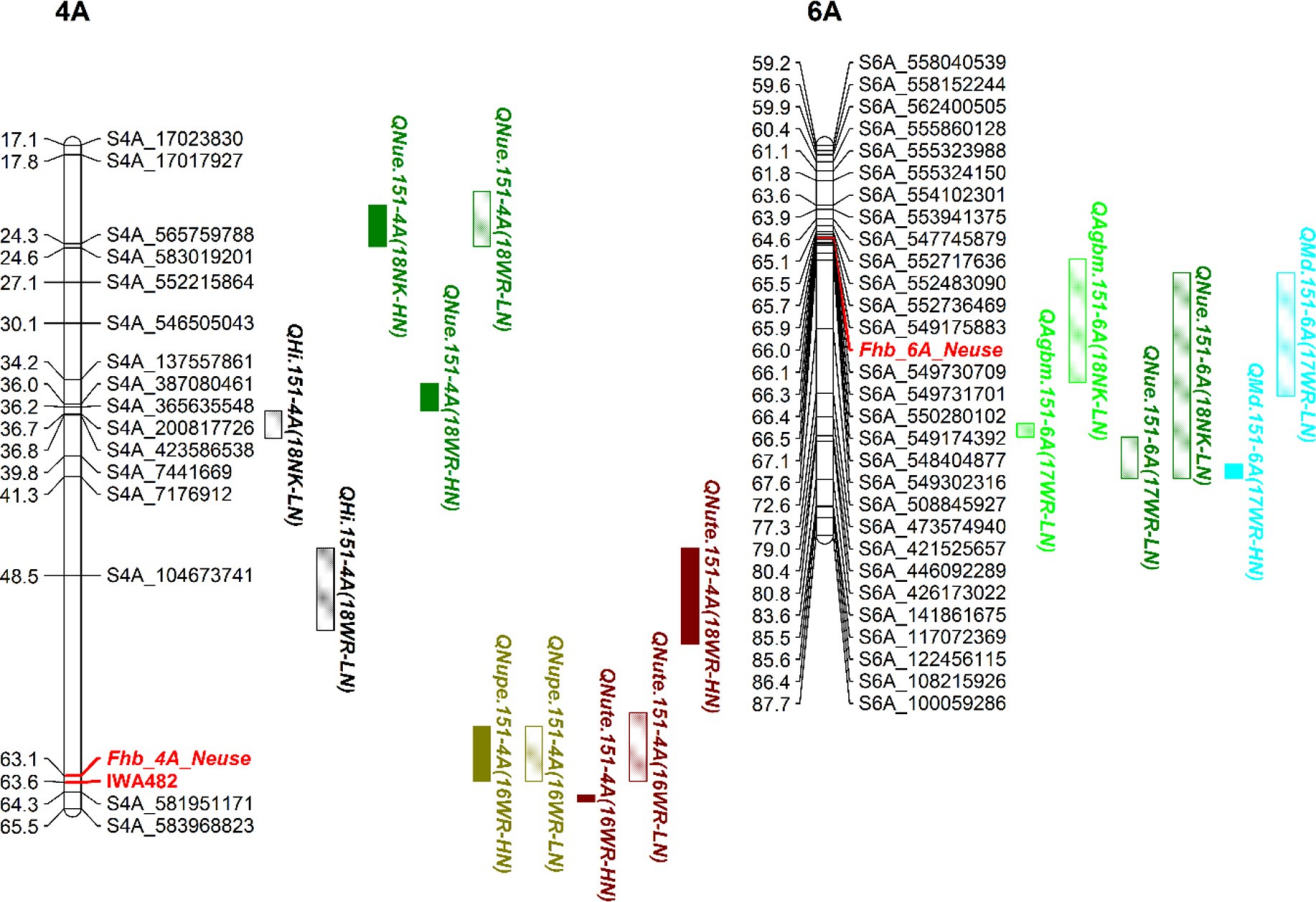
Partial linkage maps of QTL clusters on chromosomes 4A and 6A in the Yorktown × VA05W-151 wheat population. Blocks represent QTL confidence intervals, QTL colors represent traits, and the QTL fill represents QTL detected under high (solid) or low (semi-solid) N rates.

**Fig 3:**
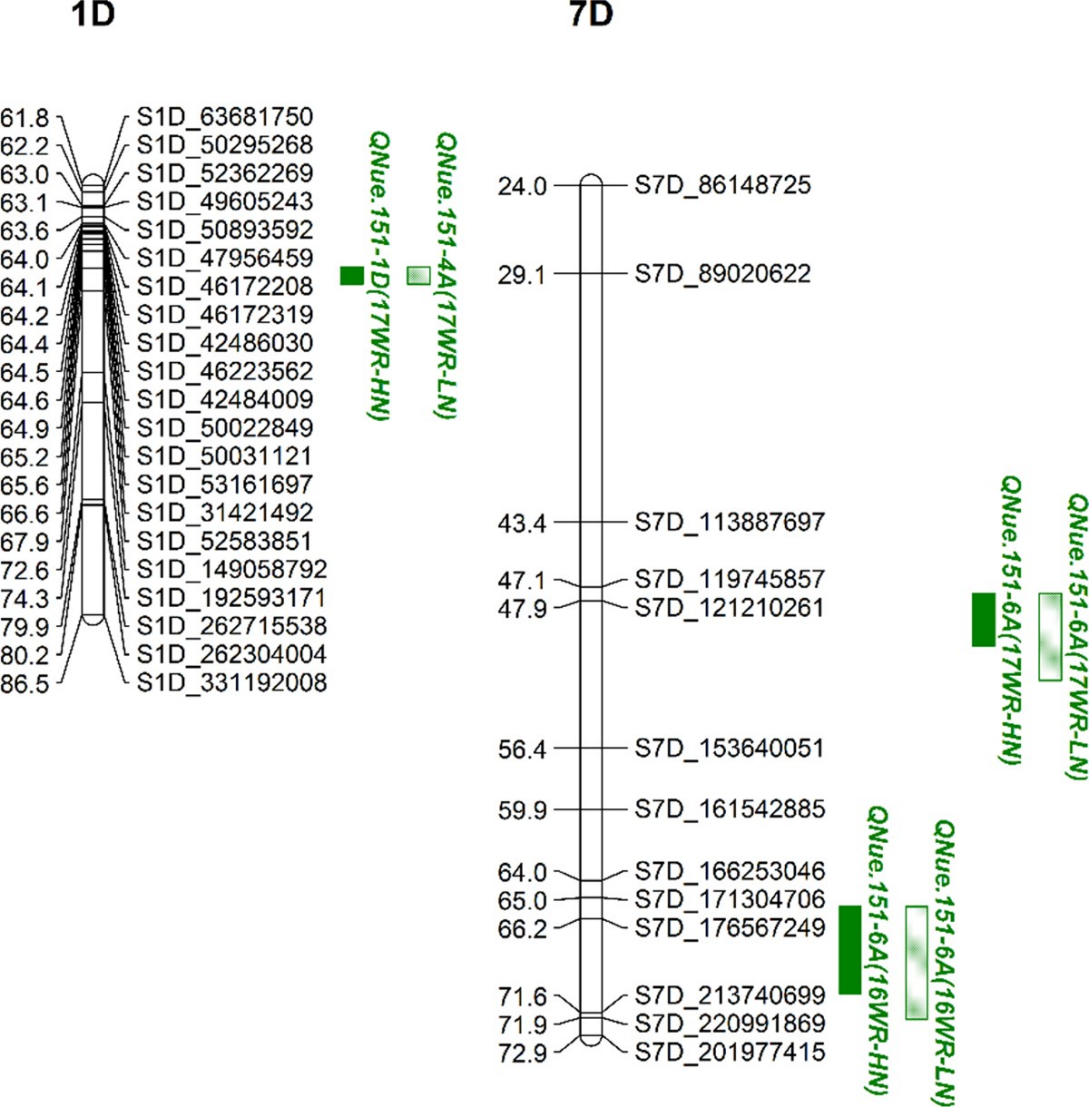
Partial linkage maps of reproducible QTL clusters on chromosomes 1D and 7D in the Yorktown × VA05W-151 wheat population. Blocks represent QTL confidence intervals, QTL colors represent traits, and the QTL fill represents QTL detected under high (solid) or low (semi-solid) N rates.

**Fig 2** illustrates partial linkage maps of the QTL on 4A and 6A. The reproducible QTL including *QNupe*.*151-4A* and *QNute*.*151-4A* clustered around the FHB 4A locus identified in ‘NC-Neuse,’ PI 633037 **[60, 63]**. *QNue*.*151-4A* and *QHi*.*151-4A* mapped to a position more than 15 cM away from the FHB-4A locus. The other QTL cluster centralized around the marker IWA4036 which is known to associate with the FHB-6A locus identified in NC-Neuse **[60]**. *QAgbm*.*151-6A*, *QNue*.*151-6A*, and *QMd*.*151-6A* were within 18 cM of the QTL for FHB resistance on 6A. Additionally, the QTL on 4A and 6A were associated with non-reproducible trait QTL (**S7 Table**). The remaining two reproducible NUE QTL (*QNue*.*151-1D* and *QNue*.*151-7D*) are illustrated in **Fig 3**. The reproducible NUE QTL on 1D co-localized with the non-reproducible QTL, *QAgbm*.*151-1D*, in the 17WR-LN environment and the reproducible NUE QTL on 7D co-localized with non-reproducible QTL, *QHgt*.*151-7D* and *QHi*.*151-7D*, in the 17WR-LN and 18WR-LN environments, respectively (**S7 Table**).

### QTL in the YT×52 population

The YT×52 population linkage maps were employed to detect QTL in the same eight N-environments as the YT×151 population. Within this population, a total of 64 QTL were identified with a LOD score greater than 2.5 (**S8 Table**) and mapped to the A (25), B (19), and D (20) genomes. However, only eight of these QTL were reproducible in two or more N-environments; these were located on chromosomes 1A, 2D, 4A, and 7A (**Table 6**). The reproducible QTL individually explained 4.9 to 52.1% of the phenotypic variation, while LOD values ranged from 2.57 for *QHgt*.*52-2D* in 18NK-LN to 26.50 for *QAd*.*52-2D* in 17WR-HN. *QNue*.*52-7A* and *QHgt*.*51-1A* were not located near any of the 116 tested SNP and SSR markers with known trait associations and explained 4.9 to 7.7% and 10.7 to 13.2% of the phenotypic variation, respectively.

**Table 6:** Quantitative trait loci (QTL) associated with N and agronomic traits in two or more N-environments in the Yorktown × VA09W-52 wheat population.

Trait	QTL	Chr.^a^	N-Env^b^	Pos.	Left marker	Right marker	LOD^c^	PVE^d^	Add^e^
								%	
AGBM^f^	*QAgbm*.*52-4A*	4A	16WR-HN	64	S352495200	S137557861	4.70	14.7	-73.15
			16WR-LN	62	S544620299	S481064477	4.40	10.0	-45.95
			18NK-LN	64	S352495200	S137557861	3.67	11.6	-57.96
NUE	*QNue*.*52-2D*	2D	16WR-HN	76	S44597217	S35039116	6.75	13.6	1.64
			16WR-LN	76	S44597217	S35039116	3.86	8.6	2.49
			17WR-HN	76	S44597217	S35039116	3.60	9.5	1.17
			17WR-LN	82	S35002830	S32151744	3.22	8.1	2.03
	*QNue*.*52-4A*	4A	16WR-HN	63	S481064477	S352495200	12.09	26.2	-2.29
			16WR-LN	63	S481064477	S352495200	8.35	20.0	-3.82
			17WR-HN	64	S352495200	S137557861	6.79	18.6	-1.64
			17WR-LN	64	S352495200	S137557861	6.55	15.7	-2.83
			18NK-HN	65	S137557861	S58223442	4.33	11.6	-1.86
			18NK-LN	64	S352495200	S137557861	8.01	23.9	-4.79
			18WR-HN	67	S58223442	S28592992	9.44	19.0	-1.74
			18WR-LN	63	S481064477	S352495200	10.75	24.0	-2.83
	*QNue*.*52-7A*	7A	16WR-HN	38	S10247569	S11056248	2.63	4.9	0.97
			16WR-LN	38	S10247569	S11056248	3.43	7.7	2.31
NUtE	*QNute*.*52-4A*	4A	18NK-HN	60	S544620299	S481064477	2.83	9.1	-0.94
			18WR-LN	63	S481064477	S352495200	2.68	6.6	-1.01
AD	*QAd*.*52-2D*	2D	16WR-HN	76	S44597217	S35039116	25.15	52.1	1.61
			16WR-LN	76	S44597217	S35039116	20.09	49.2	1.42
			17WR-HN	76	S44597217	S35039116	26.50	50.8	1.40
			17WR-LN	76	S44597217	S35039116	19.55	46.2	1.23
			18WR-HN	76	S44597217	S35039116	7.33	21.2	0.64
			18WR-LN	76	S44597217	S5039116	16.90	37.8	0.98
HGT	*QHgt*.*52-1A*	1A	18NK-HN	63	S367438005	S261248932	4.96	13.2	-2.62
			18NK-LN	63	S367438005	S261248932	3.91	10.7	-2.16
	*QHgt*.*52-2D*	2D	16WR-HN	75	S44597217	S35039116	8.83	18.4	3.24
			16WR-LN	76	S44597217	S35039116	7.89	19.8	3.28
			17WR-HN	76	S44597217	S35039116	13.85	25.1	2.68
			17WR-LN	76	S44597217	S35039116	5.18	12.8	2.31
			18NK-LN	83	S35002830	S32151744	2.57	7.6	1.83
			18WR-HN	76	S44597217	S35039116	5.27	14.9	1.87

^a^ Chromosome (Chr.).

^b^ Numbers indicate years 2015–2016 (16), 2016–2017 (17), and 2017–2018 (18); letters indicate locations Warsaw (WR) and New Kent (NK); low (LN) and high (HN) N rates within environment.

^c^ Logarithm of odds.

^d^ Percentage of phenotypic variation explained by the QTL.

^e^ Level of additivity. A positive sign indicates that alleles from Yorktown increased the trait value and a negative sign indicates that alleles from VA09W-52 increased the trait value.

^f^ Trait abbreviations for above-ground biomass (AGBM), N use efficiency (NUE), N utilization efficiency (NUtE), anthesis date (AD), and plant height (HGT). Grain yield was excluded from the results as its QTL were identical to those found for NUE.

**Fig 4:**
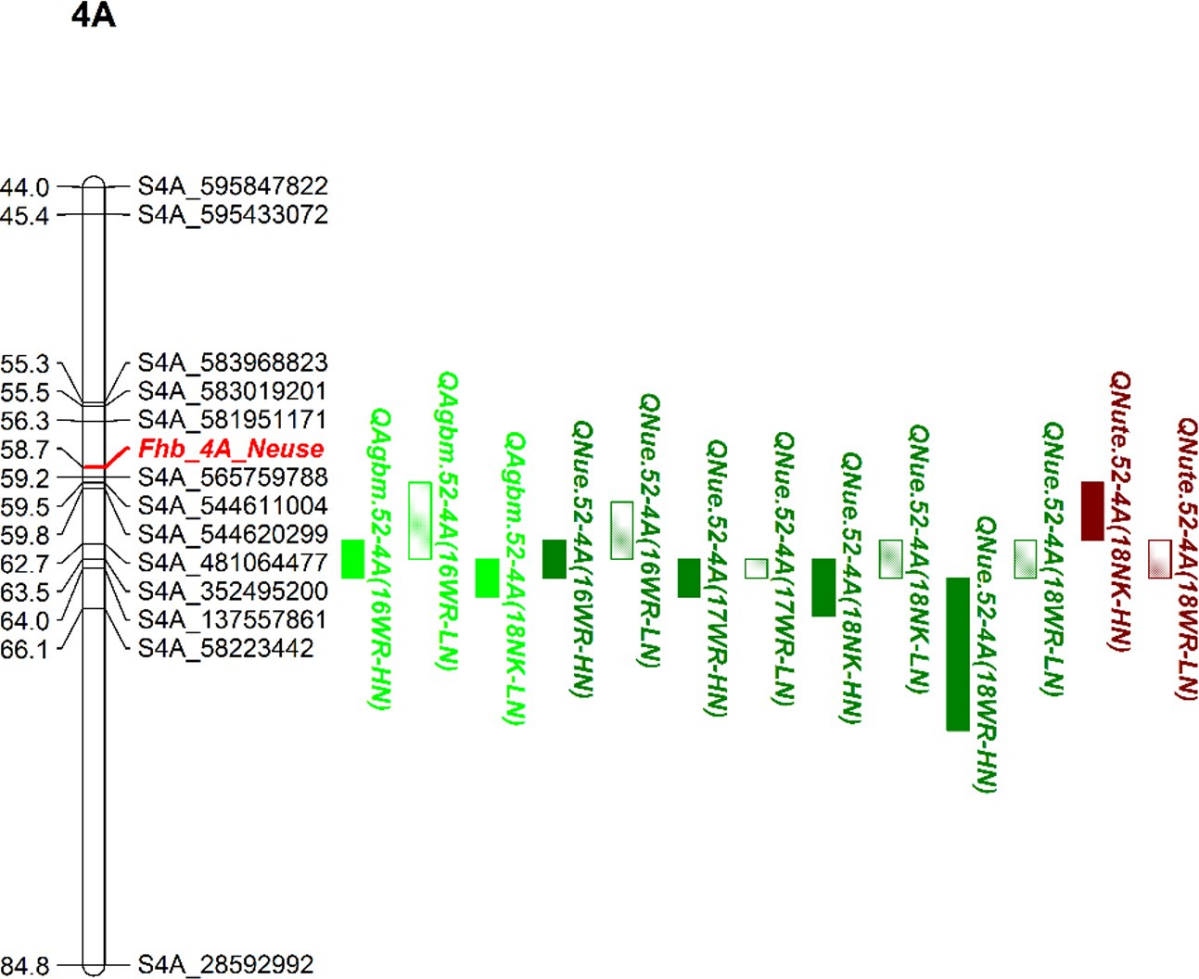
Partial linkage maps of QTL clusters on chromosome 4A in the Yorktown × VA09W-52 wheat population. Blocks represent QTL confidence intervals, QTL colors represent traits, and the QTL fill represents QTL detected under high (solid) or low (semi-solid) N rates.

**Fig 5:**
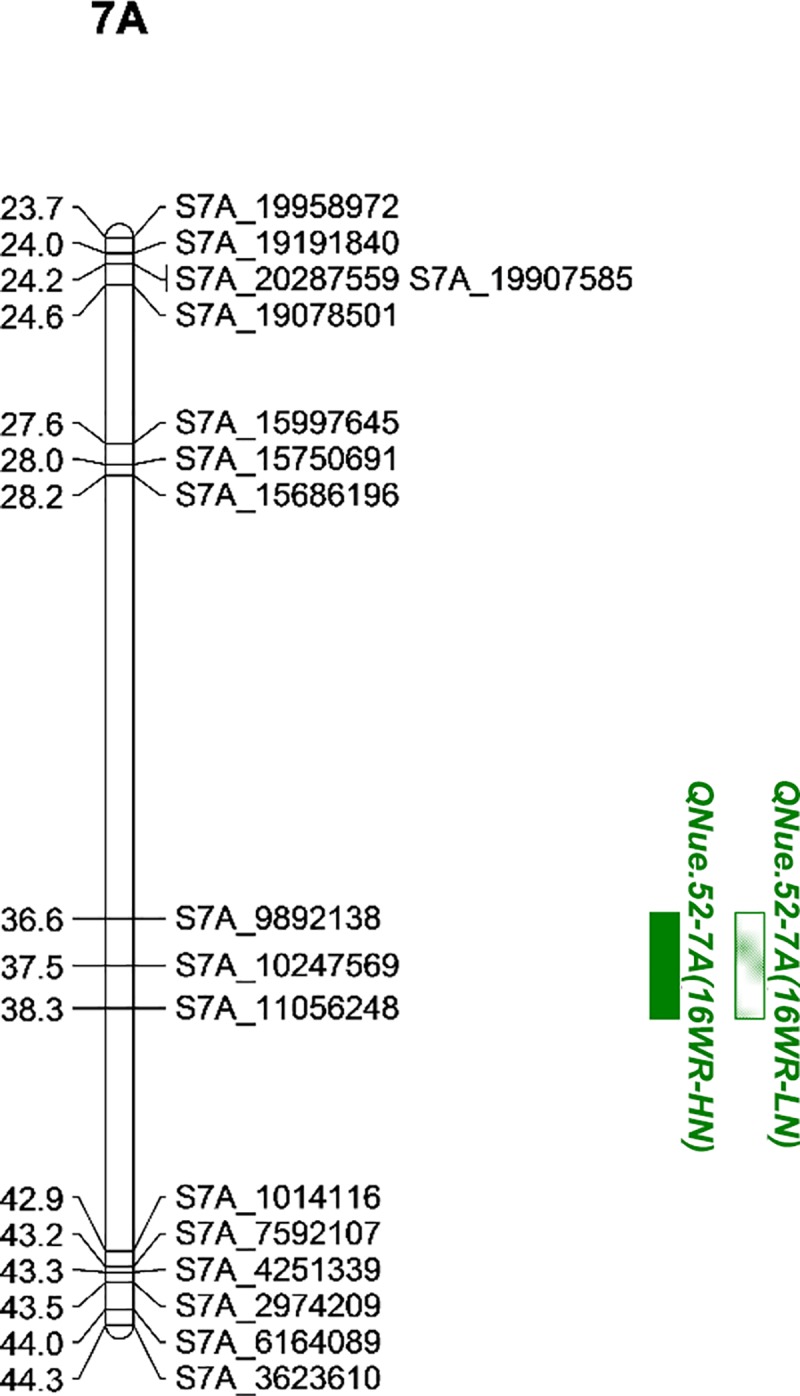
Partial linkage maps of reproducible QTL clusters on chromosome 7A in the Yorktown × VA09W-52 wheat population. Blocks represent QTL confidence intervals, QTL colors represent traits, and the QTL fill represents QTL detected under high (solid) or low (semi-solid) N rates.

**Fig 4** illustrates the reproducible QTL cluster on chromosome 4A. Similar to the results of the YT×151 population, a second QTL cluster was within 10 cM of the FHB-4A locus. The 4A cluster consisted of *QAgbm*.*vt-4A*, *QNue*.*vt-4A*, and *QNute*.*vt-4A*. A second cluster was found within 14 cM of the photoperiod response locus, Ppd-D1 **[64]**, and was comprised of *QAgbm*.*vt-2D*, *QNue*.*vt-2D*, *QAd*.*vt-2D*, and *QHgt*.*vt-2D*. Additionally, both QTL clusters in the YT×52 population were linked to QTL that were significant in only one testing environment. A third reproducible NUE QTL, *QNue*.*52-7A*, was identified in the YT×52 population (**Fig 5**) and did not co-localize with any non-reproducible QTL observed in this population (**S8 Table**).

### QTL identified in both populations

Common single and reproducible QTL identified in both the YT×151 and YT×52 populations on 1B, 2D, 3B, 3D, 4A, 5D, 6A, and 7A (**Tables 5 and 6** and **S7 and S8 Tables**). One common reproducible QTL identified in both populations was located within 10 cM of the FHB-4A locus on chromosome 4A. Additionally, the reproducible QTL found in the YT×151 population (*QHi*.*151-3B*) was located near a NUtE QTL that was identified on 3B in one N-environment (16WR-LN) in the YT×52 population. Similarly, the reproducible QTL for AGBM and NUE identified in the YT×151 population (*QAgbm*.*151-6A* and *QNue*.*151-6A*) were found in a similar position to an AGBM QTL from the YT×52 population in 16WR-LN on chromosome 6A. Six additional non-reproducible QTL that were common to both populations were identified on chromosomes 1B for NUtE (YT×151) and NUE (YT×52), 2D for grain N concentration (YT×151) and AGBM (YT×52), 3D for harvest index (YT×151) and maturity date (YT×52), 5D for grain N concentration (YT×151) and maturity date (YT×52) and on a second loci for grain N concentration (YT×151) and plant height (YT×52), and 7A for NUE in both populations.

### Effects of QTL combinations on NUE

Reproducible QTL and their combinations were assessed for NUE under LN and HN conditions. Within the YT×151 population, the presence of *QNue*.*151-1D* and *QNue*.*151-6A* significantly increased NUE over testing environments under LN by 1.8 and 1.7 kg kg^-1^, respectively (**Table 7**). The NUE QTL on 7D, *QNue*.*151-7D*, produced a significant increase in NUE under both LN (1.8 kg kg^-1^) and HN (1.2 kg kg^-1^) supplies. Nitrogen use efficiency further increased with two or three combined QTL from the YT×151 population. The YT×52 NUE QTL, *QNue*.*52-2D*, mapped near Ppd-D1 did not improve NUE under either N rate (**Table 8**). The second positive QTL, *QNue*.*52-7A*, significantly increased NUE under the reduced N rate and resulted in higher NUE than its combination with *QNue*.*52-2D*. Finally, the FHB resistance QTL on 4A was associated with decreased NUE in both populations and, therefore, this repulsion linkage will have a negative effect on improving NUE in the presence of the FHB-4A QTL. (**Tables 7 and 8**).

**Table 7:** Single and combination QTL effects on NUE (kg grain kg N^-1^) for RILs in the Yorktown ×VA05W-151 wheat population over four testing environments.

Single Loci	Low N		P.I.^a^	High N		P.I.	Combinations	Low N		P.I.	High N		P.I.
	kg kg N^-1^		%	kg kg N^-1^		%		kg kg N^-1^		%	kg kg N^-1^		%
*QNue*.*151-1D*							*1D + 6A*						
*a* (75)^b^	64.8	a^c^	2.9	36.5	a	2.2	*aa* (46)	65.5	a	4.1	36.5	a	2.5
*b* (52)	63.0	b		35.7	a		*bb* (23)	62.9	b		35.6	a	
*QNue*.*151-4A*							*1D + 7D*						
*a* (91)	63.5	b	-3.3	35.7	b	-4.2	*aa* (34)	65.7	a	5.9	37.1	a	5.7
*b* (36)	65.6	a		37.2	a		*bb* (22)	62.0	b		35.1	b	
*QNue*.*151-6A*							*6A + 7D*						
*a* (78)	64.7	a	2.7	36.2	a	0.8	*aa* (32)	66.2	a	5.6	36.7	a	4.6
*b* (48)	63.0	b		35.9	a		*bb* (19)	62.7	b		35.1	b	
*QNue*.*151-7D*							*1D + 6A + 7D*						
*a* (64)	65.1	a	2.8	36.8	a	3.3	*aaa* (19)	66.6	a	5.0	36.9	a	4.2
*b* (59)	63.3	b		35.6	b		*bbb* (9)	63.4	b		35.4	b	

^a^ Percent increase (P.I.) conferred through the ‘a’ allele.

^b^ The ‘a’ and ‘b’ alleles are inherited from Yorktown and VA05W-151, respectively. Number of individuals per allele provided in parentheses.

^c^ The LSD at *P* ≤ 0.05 is used to compare allele groupings within N rates over four testing environments; means within a single or combination of QTL followed by the same letter are not significantly different.

**Table 8:** Single and combination QTL effects on NUE (kg grain kg N^-1^) for DHs in the Yorktown × VA09W-52 population over four testing environments.

Single Loci	Low N		P.I.^a^	High N		P.I.
*QNue*.*52-2D*	kg kg N^-1^		%	kg kg N^-1^		%
*a* (83) ^b^	63.5	a^c^	1.0	35.2	a	2.0
*b* (53)	62.9	a		34.5	a	
*QNue*.*52-4A*						
*a* (56)	59.6	b	-10.2	33.0	b	-9.7
*b* (82)	65.7	a		36.2	a	
*QNue*.*52-7A*						
*a* (61)	64.3	a	3.0	35.5	a	3.2
*b* (63)	62.4	b		34.4	b	
Combination	Low N		P.I.	High N		P.I.
*2D + 7A*	kg kg N^-1^		%	kg kg N^-1^		%
*aa* (38)	64.1	a	4.7	35.5	a	5.3
*bb* (22)	61.2	b		33.7	b	

^a^ Percent increase (P.I.) conferred through the ‘a’ allele.

^b^ The ‘a’ and ‘b’ alleles are inherited from Yorktown and VA09W-52, respectively. Number of individuals per allele provided in parentheses.

^c^ The LSD at *P* ≤ 0.05 is used to compare allele groupings within N rates over four testing environments; means within a single or combination of QTL followed by the same letter are not significantly different.

## Discussion

### Trait variation and associations in the mapping populations

Transgressive segregants were identified for all traits evaluated in both mapping populations. Furthermore, trait means within N-environment were frequently intermediate of the parents, thus suggesting polygenic inheritance of a majority of the studied yield and N traits. Habash, et al. **[65]** and Fontaine, et al. **[15]** also noted non-Mendelian inheritance for NUE traits in bi-parental wheat populations grown under similar N conditions. It therefore stands to reason that the parents of the YT×151 and the YT×52 populations possess both favorable and unfavorable alleles for yield and N traits. Additional sources of beneficial NUE alleles likely exist within the eastern United States’ soft red winter wheat germplasm as genotypic variation from unrelated lineages has been reported in recent field **[30]** and greenhouse **[66]** studies of N response. Wheat lines from these panels may thus serve as a basis for future NUE mapping studies.

The present investigation decomposed grain yield into harvest index and AGBM and thereby identified a strong association between AGBM and NUE under LN and HN conditions in both populations. A similar finding was reported by Reynolds, et al. **[67]** who suggested that improving photosynthate source capacity will be required to continue yield improvement as breeders approach the theoretical maximum harvest index. In a previous investigation of 225 European wheat lines, AGBM was further shown to be highly heritable (*h*^2^ = 0.79) over environments **[9]**, but traditional phenotyping proved to be exceptionally laborious. Frels, et al. **[68]** sought to overcome this constraint by identifying high-throughput vegetative indices that were predictive of AGBM to enable more efficient phenotyping. While the investigation showed some promise for selection of genotypes expressing high AGBM, the tested vegetative indices were not consistently predictive of AGBM over growing seasons. Future work is required to improve the predictive ability of current AGBM models but the trait appears to be a suitable target for NUE breeding in winter wheat.

The present study also identified a strong relationship between NUE and its component trait, NUpE, under LN and HN rates in both populations, while the other component trait, NUtE, was only associated with NUE in the YT×52 population. Several investigations of wheat **[69–72]** previously weighed the contribution of each component trait to the variation in NUE and generally concluded that: 1) improvements in both traits are necessary to improve NUE; and 2) the influence of each trait is dependent on the environment, management practices, and genetic material being tested. It is therefore up to wheat breeders within specific regions to identify traits that most limit NUE and those for which phenotypic assessment is feasible.

### Identification of QTL for NUE traits

As expected, the GBS genotyping platform increased the number of polymorphic SNPs available in the present study compared to previous investigations that utilized the 90K-SNP array for soft red winter wheat **[73–74]**. The use of GBS increases marker density through a whole genome scan approach as opposed to screening a select number of potential polymorphisms and makes results between investigations more reproducible due to the availability of both physical and genetic positions. The present study detected similar numbers of SNPs, chromosome coverage, and marker densities as a previous bi-parental population of winter wheat that utilized GBS **[34]**. However, map length was exceptionally low on chromosome 6B in the YT×151 population which may indicate low allelic diversity on 6B between the two parents.

Previous wheat NUE mapping studies have identified QTL for N traits on every chromosome **[22, 26, 75–77]** but often lack reproducibility due to the quantitative nature of the studied traits. The low number of reproducible N-related QTL in these studies was fairly consistent with our ability to detect a large number of reproducible QTL within a single population in the present study. However, the number of reproducible QTL identified in the present investigation nearly doubles when QTL detected in a single N-environment are assessed over both populations. It therefore becomes challenging to identify which of these QTL have potential application for marker-assisted breeding programs that often employ fewer than 100 genetic markers. In response to this challenge, Quraishi, et al. **[78]** conducted a meta-analysis of QTL for NUE to identify 11 major chromosomal regions linked to N use efficiency. The NUE QTL, *QNue*.*151-6A*, identified in the present study was located near the QTL on 6A described in the aforementioned study that co-localized with a known glutamine synthetase gene (*GS*1) **[65]** and may therefore have a practical application in marker-assisted breeding due to its involvement in the assimilation of ammonium into amino acids. Additional QTL within proximity to the NUE locus on 6A were associated with NUtE and kernel weight per spike **[79]** and kernel weight **[80]** in previous studies of N response. The authors further linked this QTL on 6A to TaGW2, which influences grain size and weight **[81–82]**. The IWA4036 marker located near the reproducible QTL for NUE on chromosome 6A flanks a known FHB resistance locus **[60]**. However, the other flanking marker, IWA3483, was not segregating in the population and may therefore indicate that the genetic regions on 6A governing FHB resistance and improved NUE and N traits are different in the YT×151 population. Interestingly, this QTL was only significant under LN conditions in the YT×151 population and was also associated with AGBM under LN conditions in the YT×52 population and may therefore be a valuable marker for yield improvements under N limiting conditions.

The QTL, *QNue*.*151-1D*, inherited from Yorktown was shown to increase NUE in multiple N-environments and AGBM in one N-environment in the VA05W-151 background. Bordes, et al. **[83]** similarly identified a QTL associated with NUE in bread wheat with close proximity to the NUE QTL, *QNue*.*151-1D*, identified in the present investigation. The high-molecular-weight glutenin subunit gene, *Glu-D1*, was located more than 25 cM from this QTL and, therefore, is not likely the candidate gene **[84]**. However, Sun, et al. **[26]** reported a QTL on 1D associated with root and shoot weight in wheat seedlings grown in hydroponic culture at a similar mapping interval as the *QNue*.*151-1D* and, therefore, may indicate a role in seedling vigor. *QNue*.*52-7A* was found within the same mapping interval as QTL previously linked to grain yield and spikes per m^-2^ **[21]** and a QTL associated with NUtE and spikes per m^-2^ **[79]** under low and high N conditions. A majority of the QTL identified in the present study validated findings from previous studies, yet *QNue*.*151-7D* was not identified in marker-trait association studies for N response and may represent a novel QTL. While previous mapping studies have identified QTL within proximity to *QNue*.*151-7D*, they are primarily associated with a vernalization allele, *VRN3*, located more than 50 million nucleotides from the presently identified QTL **[19, 85, 86]**.

### QTL co-segregating with known genes

In addition to evaluation for parental variation in N response, the parents shared similar alleles for vernalization (*Vrn-A1*, *Vrn-B1*, and *Vrn-D1*), photoperiod (*Ppd-A1*, *Ppd-B1*, and *Ppd-D1*), and plant height (*Rht-B1* and *Rht-D1*). However, the *Vrn-A1* and *Ppd-D1* loci differed between Yorktown and VA09W-52 and the resulting allelic variation on 2D resulted in a strong effect QTL that explained relatively large percentages of the variation for NUE, grain yield, plant height, and anthesis date in multiple environments and N supplies. Indeed, this result is consistent with previous NUE investigations of wheat grown in the eastern **[87]** and central **[11]** United States, northwestern Mexico **[88]**, and western Europe **[9, 85]**. Worland, et al. **[89]** further observed strong interactions between testing environment and the *Ppd-D1* locus where near-isogenic lines (D1a and D1b) had increased or reduced grain yields (-16.0 to 28.4%) over a 10-year period. Similarly, the over environment effects of the *Ppd-D1* allele in the present study were not significant under LN or HN conditions despite the identification of strong effect QTL in 16WR and 17WR.

The negative effects on NUE conferred by the QTL identified near *FHB-4A* was much higher than expected upon development of the mapping populations. Indeed, associations between FHB resistance and yield *per se* is not uncommon as previous investigations found a small negative impact on grain yield **[90]** and grain quality **[91]** conferred through a FHB resistance locus on chromosome 5A. The known yield penalties associated with FHB resistance have discouraged some winter wheat breeders from utilizing exotic sources of resistance including that of spring wheat variety ‘Sumai-3’ **[92–94]**. However, the mechanisms governing a reduction in NUE by the QTL linked to FHB-4A in the present study requires further investigation to determine if this linkage can be broken.

### Implications for breeding

The QTL identified in this study that were not linked to the FHB-4A or Ppd-D1 may merit use in marker-assisted-breeding programs as they were not associated with major physiological traits or known sources of disease resistance. Genetic markers for high NUE were previously reported near *QNue*.*151-1D*, *QNue*.*151-6A*, and *QNue*.*52-7A* and, therefore, offer the most potential for immediate marker deployment. The other NUE QTL, *QNue*.*151-7D*, requires validation in unrelated populations to determine its value in NUE breeding programs. All QTL identified in this study will also benefit from introgression into diverse backgrounds to gauge their overall value to soft red winter wheat breeding programs.

## Supporting information

S1 Table Management practices in each testing environment including product name, application of fertilizer or active ingredient (A.I.), and date of application. (DOCX)

S2 Table Marker report from the Eastern Regional Small Grains Genotyping Center’s suite of 116 haplotyping markers that were screened in both wheat populations. (DOCX)

S3 Table ANOVA for agronomic and N traits of wheat parent lines Yorktown and VA05W-151 within each testing environment. (DOCX)

S4 Table ANOVA for agronomic and N traits of wheat parent lines Yorktown and VA09W-52 within each testing environment. (DOCX)

S5 Table Summary statistics of wheat parents and RILs for each trait in the Yorktown × VA05W-151 population. (DOCX)

S6 Table Summary statistics of wheat parents and DHs for each trait in the Yorktown × VA09W-52 population. (DOCX)

S7 Table Quantitative trait loci (QTL) associated with N and agronomic traits in one N-environments in the Yorktown × VA05W-151 wheat population. (DOCX)

S8 Table Quantitative trait loci (QTL) associated with N and agronomic traits in one N-environments in the Yorktown × VA09W-52 wheat population. (DOCX)

## Abbreviation

AGBM: above-ground biomass
DH: doubled haploid
FHB: Fusarium head blight
G: genotype
GBS: genotyping-by-sequencing
HN: high nitrogen rate
LN: low nitrogen rate
LOD: logarithm of odds
N: nitrogen
NK: New Kent, VA location
NUE: nitrogen use efficiency
NUpE: nitrogen uptake efficiency
NUtE: nitrogen utilization efficiency
QTL: quantitative trait loci
RIL: recombinant inbred line
SNP: single nucleotide polymorphism
SSR: simple sequence repeat
WR: Warsaw, VA location.
