# Supplementary material for: Identification of quantitative trait loci associated with nitrogen use efficiency in winter wheat
Source: PLoS One. 2020 Feb 24;15(2):e0228775. doi: 10.1371/journal.pone.0228775 (PMC7039505; doi:10.1371/journal.pone.0228775)
Supplement: S1 Table — (DOCX) [file pone.0228775.s001.docx]

**S1 Table.** Management practices in each testing environment including product name, application of fertilizer or active ingredient (A.I.), and date of application.

| Environment | Product | Application rate | Application date |
| --- | --- | --- | --- |
| 16WR | Lime | 2242 kg ha^-1^ | 09/24/2015 |
|  | Pre-plant fertilizer | 34-67-67-5S^a^ | 10/14/2015 |
|  | Planting | 480 seeds m^-2^ | 10/21/2015 |
|  | Starane™ | 0.21 kg A.I. ha^-1^ | 12/06/2015 |
|  | GS25 UAN | Variable^b^ | 02/19/2016 |
|  | Harmony Extra SG® | 0.02 kg A.I. ha^-1^ | 03/08/2016 |
|  | Starane™ | 0.15 kg A.I. ha^-1^ | 03/08/2016 |
|  | GS30 UAN | Variable | 03/12/2016 |
|  | Palisade EC | 0.06 kg A.I. ha^-1^ | 03/30/2016 |
|  | Fitness | 0.12 kg A.I. ha^-1^ | 03/30/2016 |
|  | Fitness | 0.12 kg A.I. ha^-1^ | 04/19/2016 |
|  | Prosaro | 0.14 kg A.I. ha^-1^ | 05/20/2016 |
|  | Harvest | - | 06/22/2016 |
|  |  |  |  |
| 17WR | Pre-plant fertilizer | 30-70-60-5S | 10/17/2016 |
|  | Planting | 480 seeds m^-2^ | 10/18/2016 |
|  | Quelex® | 0.01 kg A.I. ha^-1^ | 12/04/2016 |
|  | GS25 UAN | Variable | 02/06/2017 |
|  | Tilt | 0.07 kg A.I. ha^-1^ | 03/09/2017 |
|  | Tombstone | 0.03 kg A.I. ha^-1^ | 03/09/2017 |
|  | GS30 UAN | Variable | 03/13/2017 |
|  | Palisade EC | 0.08 kg A.I. ha^-1^ | 03/17/2017 |
|  | Tilt | 0.07 kg A.I. ha^-1^ | 04/05/2017 |
|  | Prosaro | 0.14 kg A.I. ha^-1^ | 04/20/2017 |
|  | Tombstone | 0.03 kg A.I. ha^-1^ | 04/28/2017 |
|  | Harvest | - | 06/12/2017 |
|  |  |  |  |
| 18WR | Lime | 2242 kg ha^-1^ | 10/04/2017 |
|  | Pre-plant fertilizer | 30-80-80-5S | 10/19/2017 |
|  | Planting | 480 seeds m^-2^ | 10/19/2017 |
|  | Harmony Extra SG® | 0.02 kg A.I. ha-1 | 11/30/2017 |
|  | Winter fertilizer | 33 kg N ha^-1^ | 12/07/2017 |
|  | GS25 UAN | Variable | 02/09/2018 |
|  | GS30 UAN | Variable | 03/10/2018 |
|  | Palisade EC | 0.08 kg A.I. ha^-1^ | 03/17/2018 |
|  | Tilt | 0.07 kg A.I. ha^-1^ | 03/17/2018 |
|  | Boron fertilizer | 0.01% (w/v) B as Na_2_B_4_O_7_ - H_2_O | 03/27/2018 |
|  | Fitness | 0.12 kg A.I. ha^-1^ | 04/11/2018 |
|  | Prosaro | 0.14 kg A.I. ha^-1^ | 05/01/2018 |
|  | Harvest | - | 06/17/2018 |
|  |  |  |  |
| 18NK | Pre-plant fertilizer | 30-50-60 | 09/26/2017 |
|  | Paraquat | 0.4 kg A.I. ha^-1^ | 10/15/2017 |
|  | Planting | 480 seeds m^-2^ | 10/22/2017 |
|  | Quelex® | 0.01 kg A.I. ha^-1^ | 12/04/2017 |
|  | Winter fertilizer | 22 kg N ha^-1^ | 12/08/2017 |
|  | Axial XL | 0.06 kg A.I. ha^-1^ | 12/18/2017 |
|  | GS25 UAN | Variable | 01/21/2018 |
|  | GS30 UAN | Variable | 02/14/2018 |
|  | Palisade EC | 0.08 kg A.I. ha^-1^ | 03/28/2018 |
|  | Prosaro | 0.14 kg A.I. ha^-1^ | 05/05/2018 |
|  | Harvest | - | 06/21/2018 |

^a^ Nitrogen (UAN), phosphorous, potassium, and sulfur re-plant fertilizer applied, respectively.

^b^ Spring N application rates split applied as 67 or 134 kg N ha^-1^.
