# Supplementary material for: Identification of quantitative trait loci associated with nitrogen use efficiency in winter wheat
Source: PLoS One. 2020 Feb 24;15(2):e0228775. doi: 10.1371/journal.pone.0228775 (PMC7039505; doi:10.1371/journal.pone.0228775)
Supplement: S2 Table — (DOCX) [file pone.0228775.s002.docx]

**S2 Table.** Marker report from the Eastern Regional Small Grains Genotyping Center’s suite of 116 haplotyping markers that were screened in both wheat populations.

| Marker name | Parent genotype | | |  | Marker name | Parent genotype | | |
| --- | --- | --- | --- | --- | --- | --- | --- | --- |
|  | YT^a^ | 52 | 151 |  |  | YT | 52 | 151 |
| KASP_cim_RhtB1_SNP | X:X | X:X | X:X |  | KASP_IWA886 | Y:Y | Y:Y | Y:Y |
| KASP_RhtD1 | Y:Y | Y:Y | Y:Y |  | KASP_IWA3805 | X:X | Y:Y | X:X |
| Rht-B1 | B1a | B1a | B1a |  | Fhb_1A_Neuse | no | 1A | no |
| Rht-D1 | D1b | D1b | D1b |  | KASP_IWA2793 | Y:Y | Y:Y | Y:Y |
| KASP_Ppd-A1prodel | Y:Y | Y:Y | Y:Y |  | KASP_IWA2900 | Y:Y | X:X | Y:Y |
| Ppd-A1 | insens | insens | insens |  | KASP_IWA402 | Y:Y | Y:Y | Y:Y |
| KASP_TaPpdBJ001 | X:X | X:X | X:X |  | KASP_IWA482 | Y:Y | X:X | X:X |
| KASP_TaPpdBJ003 | no call | no call | no call |  | Fhb_4A_Neuse | 4A | no | no |
| Ppd-B1 | ** | ** | ** |  | KASP_IWA3483 | X:X | X:X | X:X |
| KASP_TaPpdDD001 | X:X | Y:Y | X:X |  | KASP_IWA4036 | X:X | X:X | Y:Y |
| Ppd-D1 | ** | insens | ** |  | Fhb_6A_Neuse | no | no | no |
| KASP_vrn-A1_exon4 | Y:Y | X:X | Y:Y |  | KASP_IWA4606 | X:X | Y:Y | X:X |
| KASP_vrn-A1_exon7 | Y:Y | X:X | Y:Y |  | KASP_IWA5830 | X:X | X:X | X:X |
| vrn-A1 | A1 | short | A1 |  | Fhb_2B_Bess | no | no | no |
| vrn-A1_copy_number | A1 | copy | A1 |  | KASP_IWA4755 | X:X | Y:Y | Y:Y |
| KASP_5A-585397830 | Y:Y | Y:Y | Y:Y |  | KASP_IWA6381 | X:X | X:X | X:X |
| vrn-A1_MTA | short | short | short |  | KASP_IWB65344 | X:X | X:X | X:X |
| KASP_vrn-B1_AGS2K | X:X | X:X | X:X |  | Fhb_3B_Bess | no | no | no |
| vrn-B1 | B1 | B1 | B1 |  | KASP_Lr34 | X:X | X:X | X:X |
| KASP_Vrn-A1_9K001 | X:X | X:X | X:X |  | KASP_Lr34jagger | X:X | X:X | X:X |
| KASP_Vrn-A1b-Marq | X:X | X:X | X:X |  | Lr34/Yr18 | no | no | no |
| Vrn-A1 | A1 | A1 | A1 |  | KASP_Lr37_A | X:X | X:X | X:X |
| KASP_Vrn-B1_D-I | X:X | X:X | X:X |  | Yr17/Lr37/Sr38 | no | no | no |
| KASP_Vrn-B1_B | X:X | X:X | X:X |  | KASP_Lr9_A | Y:Y | no call | no call |
| KASP_Vrn-B1_C | no call | no call | no call |  | Lr9 | Lr9 | no | no |
| Vrn-B1 | B1 | B1 | B1 |  | GbFd | ** | ** | ** |
| KASP_Vrn-D1-D1a | no call | no call | no call |  | Lr19/Sr25 | no | no | no |
| Vrn-D1 | D1 | D1 | D1 |  | KASP_Sr24 | no call | no call | Y:Y |
| KASP_Fhb1 | Y:Y | Y:Y | Y:Y |  | Sr24/Lr24 | no | no | 24 |
| KASP_snp3BS-8 | X:X | X:X | X:X |  | KASP_Sr2_ger9_3p | X:X | X:X | X:X |
| Fhb1 | no | no | no |  | Sr2 | no | no | no |
| KASP_Fhb3Bc_6105 | X:X | X:X | X:X |  | KASP_Sr36_8085 | X:X | X:X | X:X |
| KASP_Fhb3Bc_8137 | X:X | X:X | X:X |  | wmc477Fd | 159 | 163 | 159 |
| Fhb_3B_Massey | no | no | no |  | Sr36/Pm6 | no | no | no |
| gwm304Fd | 198 | 198 | 198 |  | KASP_1RS_6110 | X:X | X:X | X:X |
| wmc705Pd | 160 | 160 | 162 |  | KASP_1RS_8035 | Y:Y | X:X | Y:Y |
| Fhb_5A_Ernie | no | no | no |  | KASP_IWA5194 | Y:Y | Y:Y | Y:Y |
| Fhb_5A_Ning7840 | no | no | no |  | 1RS | 1RS | no | 1RS |
| cfd233Fd | 274 | 282 | 274 |  | KASP_UN_95397731 | X:X | X:X | X:X |
| gwm539Hd | 137 | 135 | 133 |  | H13 | no | no | no |
| Fhb_2DL_Wuhan1/W14 | no | no | no |  | cfa2153Fd | ** | 203 | ** |
| KASP_IWB43992 | Y:Y | Y:Y | X:X |  | H9 | no | no | no |
| KASP_IWA6259 | Y:Y | Y:Y | X:X |  | Bdv3Fd | 184 | 184 | 184 |
| KASP_IWA7594 | X:X | X:X | X:X |  | Bdv2/3 | no | no | no |
| Fhb_1B_Jamestown | no | no | no |  | KASP_wsnp198467 | Y:Y | X:X | X:X |
| KASP_IWA1587 | Y:Y | Y:Y | Y:Y |  | Sbm1 | no | Sbm1 | Sbm1 |
| KASP_Tsn1_A | no call | no call | no call |  | Pinb-D1 | soft | soft | soft |
| Tsn1 | no | no | no |  | grain texture | soft | soft | soft |
| bx7oeFd | 400 | 400 | 400 |  | KASP_TaSus2-2B | X:X | X:X | X:X |
| Glu-B1 | no | no | no |  | sucrose synthase | no | no | no |
| umn19Fd | 341 | 359 | 341 |  | KASP_Tamyb10-A1_H | X:X | Y:Y | X:X |
| Marker name | Parent genotype | | |  | Marker name | Parent genotype | | |
|  | YT | 52 | 151 |  |  | YT | 52 | 151 |
| Glu-A1 | Ax2* | Ax1 | Ax2* |  | KASP_Tamyb10_Nor17 | X:X | X:X | X:X |
| KASP_Glu-D1d_SNP | X:X | X:X | X:X |  | Tamyb10-A1 | R-A1 | white | R-A1 |
| umn25Nd | 296 | 296 | 296 |  | KASP_Tamyb10-B1 | Y:Y | Y:Y | Y:Y |
| Glu-D1 | 2+12 | 2+12 | 2+12 |  | Tamyb10-B1 | white | white | white |
| KASP_pina-D1a | X:X | X:X | X:X |  | KASP_Tamyb10-D1 | Y:Y | Y:Y | Y:Y |
| Pina-D1 | soft | soft | soft |  | Tamyb10-D1 | R-D1 | R-D1 | R-D1 |
| KASP_pinb-wild | X:X | X:X | X:X |  | combined kernel color | RrR | rrR | RrR |

^a^ Genotypic data for Yorktown (YT), VA09W-52 (52), and VA05W-151 (151).
