# Supplementary material for: Identification of quantitative trait loci associated with nitrogen use efficiency in winter wheat
Source: PLoS One. 2020 Feb 24;15(2):e0228775. doi: 10.1371/journal.pone.0228775 (PMC7039505; doi:10.1371/journal.pone.0228775)
Supplement: S4 Table — (DOCX) [file pone.0228775.s004.docx]

**S4 Table.** ANOVA for agronomic and N traits of wheat parent lines Yorktown and VA09W-52 within each testing environment.

| Env. | Effect | YLD^a^ | GNC | AGBM | HI | AD | MD | HGT | LDG | NUE | NUpE | NUtE |
| --- | --- | --- | --- | --- | --- | --- | --- | --- | --- | --- | --- | --- |
| 16WR^b^ | G^c^ | ns^d^ | ns | ns | * | * | - | ns | ns | ns | * | * |
|  | N | * | *** | ns | ns | ns | - | ns | ns | ** | ns | ns |
|  | G × N | ns | ** | ns | ns | ns | - | ns | ns | ns | ns | ns |
| 17WR | G | ns | ns | ns | ns | ns | ns | ns | ns | ns | ns | ns |
|  | N | *** | ** | * | ns | ns | ns | ns | ns | *** | *** | *** |
|  | G × N | ns | ns | ns | ns | ns | ns | * | ns | ns | ns | ns |
| 18WR | G | ns | ns | ns | ns | ns | ns | ** | ns | ns | ns | ns |
|  | N | ns | ns | ns | ns | ns | ns | ** | ns | *** | *** | ns |
|  | G × N | ns | ns | ns | ns | ns | ns | ns | ns | ns | ns | ns |
| 18NK | G | ** | * | ns | ns | - | - | ns | ns | * | ns | ns |
|  | N | ns | ns | ns | ns | - | - | ns | ns | *** | *** | ns |
|  | G × N | ns | ns | ns | ns | - | - | ns | ns | ns | ns | ns |

^a^ Trait abbreviations for grain yield (YLD), grain N content (GNC), above-ground biomass (AGBM), harvest index (HI), anthesis date (AD), maturity date (MD), plant height (HGT), lodging (LDG), N use efficiency (NUE), N uptake efficiency (NUpE), and N utilization efficiency (NUtE).

* Significant at the 0.05 probability level.

** Significant at the 0.01 probability level.

*** Significant at the 0.001 probability level.

^b^ Numbers indicate years 2015–2016 (16), 2016-2017 (17), and 2017-2018 (18); letters indicate locations Warsaw (WR) and New Kent (NK).

^c^ G, parent genotype; N, N rate.

^d^ ns, not significant.
