# Supplementary material for: Identification of quantitative trait loci associated with nitrogen use efficiency in winter wheat
Source: PLoS One. 2020 Feb 24;15(2):e0228775. doi: 10.1371/journal.pone.0228775 (PMC7039505; doi:10.1371/journal.pone.0228775)
Supplement: S5 Table — (DOCX) [file pone.0228775.s005.docx]

**S5 Table.** Summary statistics of wheat parents and RILs for each trait in the Yorktown × VA05W-151 population.

| Trait | Env. | Parents | | | | | | | |  | RILs | | | | | |
| --- | --- | --- | --- | --- | --- | --- | --- | --- | --- | --- | --- | --- | --- | --- | --- | --- |
|  |  | Yorktown | | | | VA05W-151 | | | |  | Mean | | Range | | SD | |
|  |  | LN |  | HN |  | LN |  | HN |  |  | LN | HN | LN | HN | LN | HN |
| Grain yield  (kg ha^-1^) | 16WR | 3,907 | c^a^ | 4,901 | ab | 4,468 | b | 5,187 | a |  | 4,078 | 4,824 | 2,731–5,214 | 3,442–6,074 | 438 | 424 |
|  | 17WR | 5,059 | b | 5,947 | a | 5,392 | b | 6,159 | a |  | 5,162 | 5,980 | 4,050–6,123 | 5,255–6,928 | 365 | 379 |
|  | 18WR | 3,899 | c | 4,307 | b | 4,685 | b | 4,381 | ab |  | 4,166 | 4,264 | 2,928–5,837 | 2,999–5,410 | 383 | 483 |
|  | 18NK | 3,205 | b | 4,194 | a | 4,146 | a | 4,475 | a |  | 3,786 | 4,311 | 833–6,023 | 2,523–5,837 | 757 | 657 |
| Grain N content  (g kg^-1^) | 16WR | 1.64 | a | 1.76 | a | 1.70 | a | 1.80 | a |  | 1.95 | 2.13 | 1.39-2.63 | 1.53-2.84 | 0.35 | 0.35 |
|  | 17WR | 1.58 | ab | 1.72 | a | 1.44 | b | 1.71 | a |  | 1.45 | 1.72 | 1.10-1.85 | 1.36-2.12 | 0.15 | 0.16 |
|  | 18WR | 2.20 | b | 2.77 | a | 2.12 | b | 3.14 | a |  | 2.46 | 2.62 | 1.93-2.98 | 1.95-3.23 | 0.25 | 0.24 |
|  | 18NK | 2.54 | a | 2.65 | a | 2.29 | a | 2.40 | a |  | 2.14 | 2.80 | 1.70-3.22 | 2.22-3.87 | 0.26 | 0.35 |
| Above-ground biomass  (g m^-2^) | 16WR | 1,152 | a | 1,408 | a | 1,489 | a | 1,321 | a |  | 1,115 | 1,296 | 881–1,482 | 744–2,424 | 116 | 154 |
|  | 17WR | 1,220 | ab | 1,328 | a | 1,089 | b | 1,297 | ab |  | 1,168 | 1,313 | 957–1,408 | 1,143–1,548 | 85 | 86 |
|  | 18WR | 1,039 | c | 1,160 | b | 1,190 | b | 1,271 | a |  | 1,083 | 1,159 | 868–1,346 | 688–1,748 | 74 | 148 |
|  | 18NK | 909 | b | 1,142 | ab | 1,135 | ab | 1,245 | a |  | 1,045 | 1,157 | 148–1,718 | 681–1,644 | 224 | 165 |
| Harvest index  (g g^-1^) | 16WR | 0.34 | a | 0.35 | a | 0.31 | a | 0.39 | a |  | 0.35 | 0.38 | 0.27-0.44 | 0.20-0.61 | 0.03 | 0.04 |
|  | 17WR | 0.42 | c | 0.45 | b | 0.45 | ab | 0.47 | a |  | 0.44 | 0.46 | 0.37-0.49 | 0.36-0.50 | 0.02 | 0.02 |
|  | 18WR | 0.38 | b | 0.37 | b | 0.41 | a | 0.35 | b |  | 0.36 | 0.37 | 0.28-0.42 | 0.28-0.46 | 0.03 | 0.03 |
|  | 18NK | 0.35 | a | 0.37 | a | 0.36 | a | 0.38 | a |  | 0.39 | 0.37 | 0.28-0.45 | 0.23-0.65 | 0.03 | 0.06 |
| Anthesis date  (Julian) | 16WR | 111 | b | 111 | b | 112 | a | 112 | a |  | 111 | 111 | 108-114 | 108-115 | 1.3 | 1.2 |
|  | 17WR | 111 | a | 111 | a | 111 | a | 111 | a |  | 110 | 110 | 106-112 | 107-112 | 1.2 | 1.3 |
|  | 18WR | 120 | a | 121 | a | 120 | a | 120 | a |  | 120 | 120 | 117-123 | 117-123 | 0.9 | 1.0 |
|  | 18NK | - |  | - |  | - |  | - |  |  | - | - | - | - | - | - |
| Maturity date  (Julian) | 16WR | - |  | - |  | - |  | - |  |  | - | - | - | - | - | - |
|  | 17WR | 149 | a | 150 | a | 149 | a | 149 | a |  | 149 | 149 | 144-151 | 145-152 | 1.4 | 1.3 |
|  | 18WR | 153 | a | 153 | a | 150 | a | 150 | a |  | 150 | 151 | 149-155 | 149-157 | 1.0 | 2.0 |
|  | 18NK | - |  | - |  | - |  | - |  |  | - | - | - | - | - | - |
| Height  (cm) | 16WR | 81.3 | a | 85.2 | a | 79.2 | a | 83.1 | a |  | 81.6 | 85.1 | 68.6-95.7 | 71.6-101.3 | 5.0 | 5.2 |
|  | 17WR | 69.5 | a | 69.5 | a | 66.7 | a | 67.6 | a |  | 65.3 | 65.4 | 55.1-72.9 | 54.3-99.9 | 4.3 | 3.8 |
|  | 18WR | 87.6 | a | 87.8 | a | 86.7 | a | 88.1 | a |  | 85.7 | 87.5 | 75.4-95.7 | 76.2-99.9 | 4.1 | 4.1 |
|  | 18NK | 75.1 | a | 76.6 | a | 77.6 | a | 79.7 | a |  | 75.8 | 80.3 | 56.7-90.6 | 61.9-95.8 | 6.5 | 6.1 |
| Lodging  (0-9) | 16WR | 0 | a | 0 | a | 0 | a | 0 | a |  | 0.0 | 0.1 | 0.0-0.0 | 0.0-2.0 | 0.0 | 0.2 |
|  | 17WR | 0 | a | 0 | a | 0 | a | 0 | a |  | 0.0 | 0.0 | 0.0-0.0 | 0.0-0.0 | 0.0 | 0.0 |
|  | 18WR | 1 | b | 4 | a | 1 | b | 5 | a |  | 1.3 | 3.8 | 0.0-7.7 | 0.0-9.0 | 1.6 | 2.2 |
|  | 18NK | 0 | a | 0 | a | 0 | a | 0 | a |  | 0.2 | 1.4 | 0.0-6.0 | 0.0-7.5 | 0.8 | 1.7 |
| N-use efficiency  (kg kg^-1^) | 16WR | 58.3 | b | 36.6 | c | 66.7 | a | 38.7 | c |  | 60.9 | 36.1 | 40.8-77.8 | 25.7-45.3 | 6.6 | 3.2 |
|  | 17WR | 75.5 | b | 44.4 | c | 80.5 | a | 46.0 | c |  | 77.0 | 44.6 | 60.5-91.4 | 39.2-51.7 | 5.5 | 2.8 |
|  | 18WR | 58.1 | b | 32.1 | c | 69.9 | a | 32.7 | c |  | 62.2 | 31.8 | 43.7-78.3 | 18.8-43.6 | 5.7 | 3.6 |
|  |  | Parents | | | | | | | |  | RILs | | | | | |
| Trait | Env. | Yorktown | | | | VA05W-151 | | | |  | Mean | | Range | | SD | |
|  |  | LN |  | HN |  | LN |  | HN |  |  | LN | HN | LN | HN | LN | HN |
|  | 18NK | 47.8 | b | 31.3 | c | 61.9 | a | 33.4 | c |  | 56.0 | 32.2 | 7.70-89.9 | 22.4-40.4 | 11.9 | 4.9 |
| N-uptake efficiency  (kg kg^-1^) | 16WR | 2.84 | ab | 1.58 | b | 4.10 | a | 1.98 | b |  | 2.27 | 1.51 | 1.03-5.09 | 0.77-3.37 | 0.95 | 0.54 |
|  | 17WR | 1.74 | a | 1.07 | b | 1.58 | a | 1.12 | b |  | 1.66 | 1.16 | 1.08-3.21 | 0.84-1.79 | 0.37 | 0.21 |
|  | 18WR | 1.92 | ab | 1.38 | c | 2.12 | a | 1.59 | bc |  | 1.95 | 1.35 | 1.45-3.03 | 0.91-2.41 | 0.29 | 0.27 |
|  | 18NK | 1.89 | ab | 1.30 | b | 2.09 | a | 1.31 | b |  | 2.12 | 1.32 | 0.29-3.54 | 0.68-1.83 | 0.47 | 0.21 |
| N-utilization efficiency  (kg kg^-1^) | 16WR | 22.4 | a | 27.9 | a | 17.2 | a | 19.5 | a |  | 30.7 | 26.6 | 12.1-55.6 | 11.0-45.5 | 10.7 | 8.4 |
|  | 17WR | 44.9 | ab | 42.0 | b | 51.2 | a | 41.2 | b |  | 48.2 | 39.5 | 27.0-71.7 | 23.4-54.9 | 8.4 | 6.2 |
|  | 18WR | 30.4 | a | 23.3 | b | 33.4 | a | 21.1 | b |  | 32.3 | 24.4 | 17.8-39.9 | 17.4-30.7 | 4.1 | 4.7 |
|  | 18NK | 25.9 | b | 24.0 | b | 29.9 | a | 25.6 | b |  | 26.5 | 24.6 | 20.7-34.0 | 12.8-34.2 | 2.5 | 2.5 |

^a^ The LSD at *P* 0.05 is used to compare parental lines across N rates within an environment; means within an environment followed by the same letter are not significantly different.
