# Supplementary material for: Identification of quantitative trait loci associated with nitrogen use efficiency in winter wheat
Source: PLoS One. 2020 Feb 24;15(2):e0228775. doi: 10.1371/journal.pone.0228775 (PMC7039505; doi:10.1371/journal.pone.0228775)
Supplement: S6 Table — (DOCX) [file pone.0228775.s006.docx]

**S6 Table.** Summary statistics of wheat parents and DHs for each trait in the Yorktown × VA09W-52 population.

| Trait | Env. | Parents | | | | | | | |  | DHs | | | | | |
| --- | --- | --- | --- | --- | --- | --- | --- | --- | --- | --- | --- | --- | --- | --- | --- | --- |
|  |  | Yorktown | | | | VA09W-52 | | | |  | Mean | | Range | | SD | |
|  |  | LN |  | HN |  | LN |  | HN |  |  | LN | HN | LN | HN | LN | HN |
| Grain yield  (kg ha^-1^) | 16WR | 3,659 | c^a^ | 4,867 | a | 4,068 | bc | 4,607 | ab |  | 3,837 | 4,670 | 2,407–5,067 | 3,216–6,098 | 542 | 562 |
|  | 17WR | 4,924 | b | 5,832 | a | 5,092 | b | 6,106 | a |  | 4,876 | 5,730 | 3,750–6,151 | 3,937–6,834 | 450 | 500 |
|  | 18WR | 4,120 | ab | 3,883 | b | 4,247 | a | 4,027 | ab |  | 3,864 | 3,936 | 2,756–4,912 | 2,111–5,139 | 408 | 548 |
|  | 18NK | 4,250 | b | 4,038 | b | 5,081 | a | 4,950 | a |  | 4,372 | 4,372 | 2,355–5,717 | 2,428–6,014 | 650 | 741 |
| Grain N content  (g kg^-1^) | 16WR | 1.69 | c | 1.89 | b | 1.51 | d | 2.03 | a |  | 1.67 | 1.90 | 1.27-2.02 | 1.57-2.43 | 0.16 | 0.15 |
|  | 17WR | 1.68 | b | 2.11 | a | 1.57 | b | 1.94 | a |  | 1.72 | 2.04 | 1.28-2.49 | 1.52-2.66 | 0.16 | 0.19 |
|  | 18WR | 2.56 | a | 2.59 | a | 2.71 | a | 2.98 | a |  | 2.39 | 2.79 | 1.81-3.20 | 2.11-3.49 | 0.29 | 0.26 |
|  | 18NK | 2.54 | ab | 2.61 | a | 2.45 | b | 2.48 | ab |  | 2.46 | 2.55 | 1.97-2.96 | 1.98-3.06 | 0.20 | 0.19 |
| Above-ground biomass  (g m^-2^) | 16WR | 1,101 | ab | 1,306 | a | 910 | bc | 1,076 | ab |  | 1,004 | 1,165 | 555–1,487 | 726–2,343 | 140 | 191 |
|  | 17WR | 1,108 | b | 1,239 | ab | 1,119 | bc | 1,325 | a |  | 1,129 | 1,257 | 860–1,531 | 342–1,777 | 138 | 150 |
|  | 18WR | 1,299 | a | 1,107 | a | 1,124 | a | 1,186 | a |  | 1,085 | 1,112 | 737–1,682 | 619–1,673 | 156 | 148 |
|  | 18NK | 1,156 | a | 968 | a | 1,204 | a | 1,092 | a |  | 1,089 | 1,108 | 628–1,475 | 541–1,507 | 168 | 193 |
| Harvest index  (g g^-1^) | 16WR | 0.33 | c | 0.37 | bc | 0.45 | a | 0.43 | ab |  | 0.38 | 0.40 | 0.30-0.48 | 0.24-0.58 | 0.04 | 0.04 |
|  | 17WR | 0.46 | a | 0.47 | a | 0.46 | a | 0.47 | a |  | 0.44 | 0.46 | 0.30-0.52 | 0.28-0.64 | 0.04 | 0.04 |
|  | 18WR | 0.33 | a | 0.35 | a | 0.38 | a | 0.34 | a |  | 0.36 | 0.35 | 0.21-0.52 | 0.21-0.49 | 0.04 | 0.04 |
|  | 18NK | 0.37 | a | 0.43 | a | 0.42 | a | 0.46 | a |  | 0.40 | 0.40 | 0.30-0.49 | 0.30-0.55 | 0.03 | 0.05 |
| Anthesis date  (Julian) | 16WR | 111 | a | 112 | a | 108 | a | 108 | a |  | 112 | 112 | 108-117 | 107-117 | 2.1 | 2.2 |
|  | 17WR | 109 | a | 109 | a | 109 | a | 109 | a |  | 110 | 110 | 106-113 | 106-115 | 1.8 | 1.9 |
|  | 18WR | 121 | a | 120 | a | 120 | a | 120 | a |  | 121 | 121 | 118-125 | 116-124 | 1.6 | 1.5 |
|  | 18NK | - |  | - |  | - |  | - |  |  | - | - | - | - | - | - |
| Maturity date  (Julian) | 16WR | - |  | - |  | - |  | - |  |  | - | - | - | - | - | - |
|  | 17WR | 149 | a | 149 | a | 150 | a | 150 | a |  | 149 | 149 | 145-152 | 146-153 | 1.6 | 1.3 |
|  | 18WR | 153 | a | 152 | a | 150 | a | 152 | a |  | 151 | 152 | 149-156 | 148-158 | 1.7 | 2.5 |
|  | 18NK | - |  | - |  | - |  | - |  |  | - | - | - | - | - | - |
| Height  (cm) | 16WR | 78.8 | a | 87.5 | a | 81.0 | a | 84.1 | a |  | 82.0 | 86.4 | 60.6-98.7 | 64.7-101.1 | 7.2 | 6.5 |
|  | 17WR | 69.1 | a | 65.3 | a | 65.7 | a | 68.7 | a |  | 68.8 | 69.3 | 49.9-89.7 | 57.1-85.0 | 5.7 | 5.1 |
|  | 18WR | 82.0 | b | 88.8 | a | 88.8 | a | 91.3 | a |  | 87.1 | 88.1 | 70.7-98.6 | 71.8-104.0 | 5.5 | 5.0 |
|  | 18NK | 82.4 | a | 83.8 | a | 81.6 | a | 83.5 | a |  | 84.2 | 87.5 | 71.4-102.7 | 73.9-107.0 | 6.5 | 6.7 |
| Lodging  (0-9) | 16WR | 0 | a | 0 | a | 0 | a | 0 | a |  | 0 | 0.0 | 0.0-1.0 | 0.0-6.0 | 0.1 | 0.6 |
|  | 17WR | 0 | a | 0 | a | 0 | a | 0 | a |  | 0 | 0.0 | 0.0-0.0 | 0.0-0.0 | 0.0 | 0.0 |
|  | 18WR | 2 | b | 3 | ab | 4 | ab | 6 | a |  | 1.4 | 4.2 | 0.0-6.3 | 0.0-9.0 | 1.5 | 2.7 |
|  | 18NK | 0 | a | 0 | a | 0 | a | 1 | a |  | 0.4 | 1.3 | 0.0-5.8 | 0.0-7.1 | 0.8 | 1.6 |
| N-use efficiency  (kg kg^-1^) | 16WR | 54.6 | a | 36.3 | b | 60.7 | a | 34.4 | b |  | 57.2 | 34.9 | 35.9-75.6 | 24.0-45.5 | 8.1 | 4.2 |
|  | 17WR | 73.5 | a | 43.5 | b | 76.0 | a | 45.6 | b |  | 72.8 | 65.3 | 56.0-91.8 | 35.1-85.3 | 6.7 | 9.7 |
|  | 18WR | 61.5 | a | 29.0 | b | 63.4 | a | 30.4 | b |  | 57.7 | 29.4 | 41.1-73.3 | 15.8-38.4 | 6.1 | 4.1 |
| Trait | Env. | Parents | | | | | | | |  | DHs | | | | | |
|  |  | Yorktown | | | | VA09W-52 | | | |  | Mean | | Range | | SD | |
|  |  | LN |  | HN |  | LN |  | HN |  |  | LN | HN | LN | HN | LN | HN |
|  | 18NK | 63.4 | b | 30.1 | c | 74.5 | a | 36.9 | c |  | 65.3 | 32.6 | 35.1-85.3 | 18.1-44.9 | 9.7 | 5.3 |
| N-uptake efficiency  (kg kg^-1^) | 16WR | 2.66 | a | 1.44 | b | 1.27 | b | 1.01 | b |  | 1.66 | 1.12 | 0.98-3.80 | 0.67-2.23 | 0.62 | 0.31 |
|  | 17WR | 1.63 | a | 1.21 | b | 1.56 | a | 1.23 | b |  | 1.68 | 2.42 | 1.26-2.63 | 1.50-3.73 | 0.20 | 0.41 |
|  | 18WR | 2.77 | a | 1.25 | b | 2.60 | a | 1.53 | b |  | 2.11 | 1.38 | 1.40-3.54 | 0.85-4.64 | 0.33 | 0.36 |
|  | 18NK | 2.62 | a | 1.22 | b | 2.69 | a | 1.30 | b |  | 2.42 | 1.31 | 1.50-3.73 | 0.71-1.73 | 0.41 | 0.21 |
| N-utilization efficiency  (kg kg^-1^) | 16WR | 21.7 | b | 27.8 | b | 48.0 | a | 34.2 | ab |  | 38.5 | 32.9 | 16.7-59.0 | 17.3-47.6 | 10.1 | 7.2 |
|  | 17WR | 45.1 | a | 36.0 | b | 48.7 | a | 37.3 | b |  | 42.8 | 27.2 | 29.4-51.0 | 20.6-35.7 | 3.7 | 2.9 |
|  | 18WR | 22.3 | a | 23.7 | a | 25.6 | a | 20.0 | a |  | 27.8 | 22.0 | 17.5-39.3 | 7.1-33.4 | 4.1 | 3.7 |
|  | 18NK | 24.3 | a | 25.2 | a | 27.7 | a | 28.5 | a |  | 27.2 | 25.1 | 20.6-35.7 | 18.7-33.3 | 2.9 | 3.1 |

^a^ The LSD at *P* 0.05 is used to compare parental lines across N rates within an environment; means within an environment followed by the same letter are not significantly different.
