# Supplementary material for: Identification of quantitative trait loci associated with nitrogen use efficiency in winter wheat
Source: PLoS One. 2020 Feb 24;15(2):e0228775. doi: 10.1371/journal.pone.0228775 (PMC7039505; doi:10.1371/journal.pone.0228775)
Supplement: S8 Table — (DOCX) [file pone.0228775.s008.docx]

**S8 Table.** Quantitative trait loci (QTL) associated with N and agronomic traits in one N-environments in the Yorktown × VA09W-52 wheat population.

| Trait | Chr.^a^ | N-Env.^b^ | Pos. | Left marker | Right marker | LOD^c^ | PVE^d^ | Add^e^ |
| --- | --- | --- | --- | --- | --- | --- | --- | --- |
|  |  |  |  |  |  |  | % |  |
| AD^f^ | 1B | 17WR-HN | 89 | S563121178 | S562485837 | 2.81 | 3.47 | 0.36 |
|  | 4A | 17WR-HN | 125 | S7176912 | S2786681 | 4.42 | 6.06 | -0.48 |
|  | 4A | 18WR-LN | 61 | S544620299 | S481064477 | 4.73 | 8.60 | 0.47 |
|  | 5A | 16WR-HN | 73 | S596570258 | S595425852 | 3.28 | 4.74 | 0.48 |
|  | 5A | 17WR-LN | 99 | S567114202 | S564579859 | 3.71 | 6.88 | -0.46 |
|  | 5D | 17WR-HN | 68 | S601232900 | S598810451 | 3.53 | 4.42 | -0.40 |
|  | 7B | 16WR-LN | 31 | S19536241 | S34848225 | 2.51 | 4.39 | -0.42 |
| AGBM | 2D | 17WR-LN | 76 | S44597217 | S35039116 | 2.84 | 7.81 | 40.77 |
|  | 3B | 16WR-LN | 84 | S39455478 | S29498148 | 3.06 | 8.48 | -41.34 |
|  | 5B | 16WR-LN | 153 | S670064794 | S669690464 | 4.15 | 9.41 | 43.55 |
|  | 6A | 16WR-LN | 3 | S52019595 | S51375303 | 4.15 | 9.32 | 43.4 |
| GNC | 5A | 16WR-HN | 208 | S9395730 | S7493203 | 2.99 | 11.74 | 0.05 |
|  | 5A | 18WR-LN | 157 | S458562272 | S455344314 | 3.88 | 10.18 | 0.10 |
|  | 7B | 18NK-LN | 15 | S707714032 | S706277402 | 2.85 | 8.33 | -0.06 |
|  | 7D | 17WR-HN | 31 | S527727944 | S524324303 | 3.31 | 10.46 | 0.06 |
| HGT | 1A | 17WR-HN | 52 | S497201595 | S478424706 | 4.34 | 6.84 | -1.38 |
|  | 2B | 18WR-HN | 21 | S775486182 | S775329395 | 3.19 | 8.72 | 1.41 |
|  | 3B | 16WR-HN | 82 | S39455478 | S29498148 | 2.90 | 6.28 | 1.86 |
|  | 4A | 16WR-HN | 49 | S595433072 | S583968823 | 3.71 | 7.60 | -2.06 |
|  | 5A | 18NK-HN | 95 | S567502385 | S567534925 | 4.28 | 11.09 | -2.35 |
|  | 5A | 18WR-HN | 82 | S585018041 | S584664469 | 2.68 | 7.11 | -1.27 |
|  | 5D | 17WR-LN | 112 | S396986910 | S63267701 | 2.75 | 10.34 | 2.04 |
|  | 7B | 16WR-HN | 71 | S210333999 | S483533955 | 5.30 | 9.80 | -2.32 |
|  | 7D | 17WR-HN | 75 | S86148725 | S73368696 | 4.52 | 6.91 | 1.38 |
|  | 7D | 17WR-HN | 101 | S55405801 | S53710322 | 8.43 | 13.76 | -1.95 |
|  | 7D | 17WR-HN | 0 | S5068391 | S15199996 | 3.01 | 4.44 | -1.11 |
| HI | 2A | 18NK-LN | 3 | S25669457 | S22128148 | 4.19 | 11.36 | 0.01 |
|  | 2B | 17WR-LN | 0 | S67433042 | S67431792 | 2.61 | 8.65 | 0.01 |
|  | 4A | 18NK-HN | 66 | S137557861 | S58223442 | 5.13 | 15.92 | -0.02 |
|  | 4A | 18WR-LN | 87 | S28592992 | S24753888 | 3.24 | 8.12 | -0.01 |
|  | 4B | 18WR-LN | 75 | S88225322 | S31876652 | 6.28 | 16.48 | -0.02 |
| LDG | 2B | 18WR-LN | 53 | S707177604 | S705673880 | 2.75 | 8.90 | 0.43 |
|  | 2D | 18NK-HN | 41 | S75260724 | S75756585 | 2.93 | 9.66 | 0.50 |
|  | 3B | 18WR-HN | 23 | S647967657 | S602479682 | 4.34 | 11.96 | 0.97 |
|  | 5D | 18WR-HN | 95 | S409542642 | S396986910 | 2.66 | 7.89 | 0.78 |
| MD | 3D | 17WR-LN | 42 | S460249827 | S431289828 | 5.26 | 14.13 | -0.69 |
|  | 4D | 17WR-LN | 0 | S456054449 | S455763126 | 3.50 | 7.30 | 0.50 |
|  | 5D | 17WR-HN | 85 | S421533203 | S409542642 | 2.75 | 7.39 | -0.39 |
|  | 5D | 18WR-HN | 132 | S254497728 | S251953300 | 3.09 | 7.96 | 0.73 |
|  | 7B | 18WR-HN | 71 | S210333999 | S483533955 | 3.39 | 9.05 | 0.77 |
|  | 7D | 17WR-HN | 31 | S527727944 | S524324303 | 3.65 | 9.66 | -0.44 |
| NUE | 1A | 18NK-HN | 56 | S475625631 | S394817972 | 2.54 | 6.80 | 1.41 |
|  | 1B | 18NK-HN | 23 | S656423128 | S655908290 | 2.59 | 6.61 | 1.40 |
|  | 3A | 18WR-HN | 75 | S571169985 | S568645681 | 6.58 | 12.21 | 1.38 |
|  | 3B | 18WR-HN | 92 | S27484145 | S27493643 | 2.62 | 4.50 | 0.83 |
|  | 5A | 18WR-LN | 157 | S458562272 | S455344314 | 2.53 | 5.13 | -1.27 |
|  | 7A | 18WR-HN | 35 | S112439521 | S111912514 | 4.19 | 7.5 | 1.08 |
| NUpE | 1A | 18NK-HN | 55 | S464836988 | S475625631 | 3.07 | 8.88 | 0.07 |
|  | 2D | 18NK-HN | 5 | S556333501 | S442052650 | 2.88 | 8.94 | 0.07 |
|  | 7B | 18NK-LN | 15 | S707714032 | S706277402 | 3.66 | 11.48 | -0.14 |
| NUtE | 2A | 18NK-LN | 3 | S25669457 | S22128148 | 3.00 | 9.65 | 0.91 |
| Trait | Chr. | N-Env. | Pos. | Left marker | Right marker | LOD | PVE | Add |
|  |  |  |  |  |  |  | % |  |
|  | 3B | 16WR-LN | 65 | S45271380 | S44274197 | 2.53 | 8.08 | -2.84 |
|  | 3B | 18WR-LN | 30 | S586509678 | S523569541 | 2.78 | 6.90 | -1.01 |
|  | 3B | 18WR-LN | 42 | S762639074 | S760963490 | 2.73 | 6.81 | -1.00 |
|  | 5A | 18WR-LN | 157 | S458562272 | S455344314 | 3.96 | 10.37 | -1.23 |
|  | 7D | 17WR-HN | 32 | S529393082 | S529349312 | 4.37 | 13.80 | -1.70 |
|  | 7D | 18WR-LN | 85 | S86148725 | S73368696 | 3.26 | 8.17 | -1.10 |

^a^ Chromosome (Chr.).

^b^ Numbers indicate years 2015–2016 (16), 2016-2017 (17), and 2017-2018 (18); letters indicate locations Warsaw (WR) and New Kent (NK); low (LN) and high (HN) N rates within environment.

^c^ Logarithm of odds.

^d^ Percentage of phenotypic variation explained by the QTL.

^e^ Level of additivity. A positive sign indicates that alleles from Yorktown increased the trait value and a negative sign indicates that alleles from VA09W-52 increased the trait value.

^f^ Trait abbreviations for grain N content (GNC), above-ground biomass (AGBM), harvest index (HI), N use efficiency (NUE), N uptake efficiency (NUpE), N utilization efficiency (NUtE), anthesis date (AD), maturity date (MD), plant height (HGT), and lodging (LDG). Grain yield was excluded from the results as its QTL were identical to those found for NUE.
